# Predicting gait adaptations due to ankle plantarflexor muscle weakness and contracture using physics-based musculoskeletal simulations

**DOI:** 10.1371/journal.pcbi.1006993

**Published:** 2019-10-07

**Authors:** Carmichael F. Ong, Thomas Geijtenbeek, Jennifer L. Hicks, Scott L. Delp

**Affiliations:** 1 Department of Bioengineering, Stanford University, Stanford, California, United States of America; 2 Department of Biomechatronics & Human-Machine Control, Delft University of Technology, Delft, The Netherlands; 3 Department of Mechanical Engineering, Stanford University, Stanford, California, United States of America; 4 Department of Orthopaedic Surgery, Stanford University, Stanford, California, United States of America; The Ohio State University, UNITED STATES

## Abstract

Deficits in the ankle plantarflexor muscles, such as weakness and contracture, occur commonly in conditions such as cerebral palsy, stroke, muscular dystrophy, Charcot-Marie-Tooth disease, and sarcopenia. While these deficits likely contribute to observed gait pathologies, determining cause-effect relationships is difficult due to the often co-occurring biomechanical and neural deficits. To elucidate the effects of weakness and contracture, we systematically introduced isolated deficits into a musculoskeletal model and generated simulations of walking to predict gait adaptations due to these deficits. We trained a planar model containing 9 degrees of freedom and 18 musculotendon actuators to walk using a custom optimization framework through which we imposed simple objectives, such as minimizing cost of transport while avoiding falling and injury, and maintaining head stability. We first generated gaits at prescribed speeds between 0.50 m/s and 2.00 m/s that reproduced experimentally observed kinematic, kinetic, and metabolic trends for walking. We then generated a gait at self-selected walking speed; quantitative comparisons between our simulation and experimental data for joint angles, joint moments, and ground reaction forces showed root-mean-squared errors of less than 1.6 standard deviations and normalized cross-correlations above 0.8 except for knee joint moment trajectories. Finally, we applied mild, moderate, and severe levels of muscle weakness or contracture to either the soleus (SOL) or gastrocnemius (GAS) or both of these major plantarflexors (PF) and retrained the model to walk at a self-selected speed. The model was robust to all deficits, finding a stable gait in all cases. Severe PF weakness caused the model to adopt a slower, "heel-walking" gait. Severe contracture of only SOL or both PF yielded similar results: the model adopted a "toe-walking" gait with excessive hip and knee flexion during stance. These results highlight how plantarflexor weakness and contracture may contribute to observed gait patterns.

## Author summary

Deficits in the ankle plantarflexors, which are muscles that extend the ankle, are thought to contribute to abnormal walking patterns in conditions such as cerebral palsy, stroke, muscular dystrophy, Charcot-Marie-Tooth disease, and aging. To study how deficits in these muscles contribute to abnormal walking patterns, we used computer simulations to systematically introduce muscle deficits into a biomechanically accurate model. We first showed that our model could discover realistic walking patterns over a wide range of speeds when we posed a simple objective: walking while consuming a minimum amount of energy per distance, maintaining head stability, and avoiding injury. We then used the model to study the effect of two commonly observed problems: muscle weakness and muscle tightness. We found that severe weakness of the ankle plantarflexors caused the model to adopt a slower, “heel-walking” gait, and severe tightness caused the model to adopt a crouched, “toe-walking” gait. These results highlight how deficits in the ankle plantarflexors muscles may contribute to abnormal walking patterns commonly seen in pathological populations.

## Introduction

The ankle plantarflexor muscles play an important role in human walking, and deficits in these muscles are thought to contribute to gait pathologies. Previous work has shown that the plantarflexor muscles are important for generating forward acceleration during the push-off phase of walking [1,2]. Individuals with cerebral palsy, stroke, muscular dystrophy, or Charcot-Marie-Tooth disease often exhibit plantarflexor muscle weakness [3–9] and contracture [3,9–15]. Sarcopenia, or muscle loss due to aging, affects almost half of the population by age 75 and is also characterized by plantarflexor muscle weakness [16]. However, these muscular deficits often co-occur with other abnormalities, such as skeletal deformities and neural deficits, making it difficult to assess the independent contributions of muscular deficits to gait abnormalities.

Previous experimental work has sought to better understand the cause-effect relationship between plantarflexor weakness and gait pathologies. In one study, a tibial-nerve block was performed in one leg in healthy subjects [17]. Adaptations on the affected side included a reduced ability to transfer weight to the contralateral leading limb during terminal stance and a shortened single-limb stance duration. Although this experiment helps to establish a cause-effect relationship, nerve blocks are invasive and thus difficult to repeat for many muscles. Further, these experiments cannot reveal the effects of contracture, which produces abnormally high passive stiffness of muscles.

Musculoskeletal simulations built from experimental gait data have been used to study gait pathologies. For example, simulations of individuals with cerebral palsy have quantified individual muscle contributions to body weight support and forward propulsion [18], the minimum muscle strength required to walk in a crouch gait [19], and the contributions of contracture and spasticity to increased hamstring resistance [20]. These studies suggest strong links between muscle deficits and the observed gait adaptations; however, since these studies tracked experimental data from patients with a combination of muscular, skeletal, and neural deficits, the independent effects of muscular weakness and contracture on the observed gait adaptations cannot be assessed.

Simulations in which kinematics are generated *de novo* (i.e., without tracking experimental data) can help reveal cause-effect relationships between muscular deficits and gait abnormalities. Researchers have created reflex-based controllers using single shooting trajectory optimization that could generate various gait patterns, including level walking [21–24] and running [22,24], inclined walking [23,24], loaded walking [23], stair ascent, and turning [24]. Other trajectory optimization methods, such as multiple shooting and direct collocation, have been used to generate simulations of walking [25] and running [26] and to study prostheses [27,28]. Recently, Song and colleagues used a reflex-based controller and single shooting approach to understand which factors contributed to decreased walking performance in the elderly [29]. By systematically introducing the neural, muscular, or skeletal deficits seen in this population and training each model to walk, they determined that decreased muscle strength and mass of all muscles contributed most to typically observed gait adaptations. Although simulations of this type are valuable, they are not yet broadly used because it is challenging to generate simulations and to reproduce previous work. Overcoming these challenges has been difficult because of the sensitivity of forward simulations to a software environment and limited sharing of software and models. More work is also needed to validate that simulations of this type capture the adaptations observed in experiments (e.g., due to varying walking speeds), before adjusting the model to generate simulations under new conditions that cannot be observed in controlled experiments.

The goal of our work was to determine which gait adaptations arise from weakness or contracture of the plantarflexor muscles. To this end, we first created and validated an optimization framework and musculoskeletal model that could generate realistic motions *de novo*. Our controller followed the previously described reflex-based controllers [21,22,24], and the parameters of our controller were iteratively updated within the optimization framework. We validated our results over a wide range of prescribed walking speeds and at self-selected speed; this step was necessary as low gait speed is commonly observed in individuals with gait impairments. We then introduced weakness or contracture in the plantarflexor muscles, generated new gait patterns, and analyzed subsequent changes in the kinematics and kinetics of gait. Our model and results are freely available at https://simtk.org/projects/pfdeficitsgait along with a Docker build file and setup files so others can reproduce our work. We also provide downloads for the control and optimization framework (SCONE, Simulated Controller OptimizatioN Environment [30]) and accompanying documentation at https://scone.software to enable others to use and build upon our simulation framework.

## Methods

**Fig 1:**
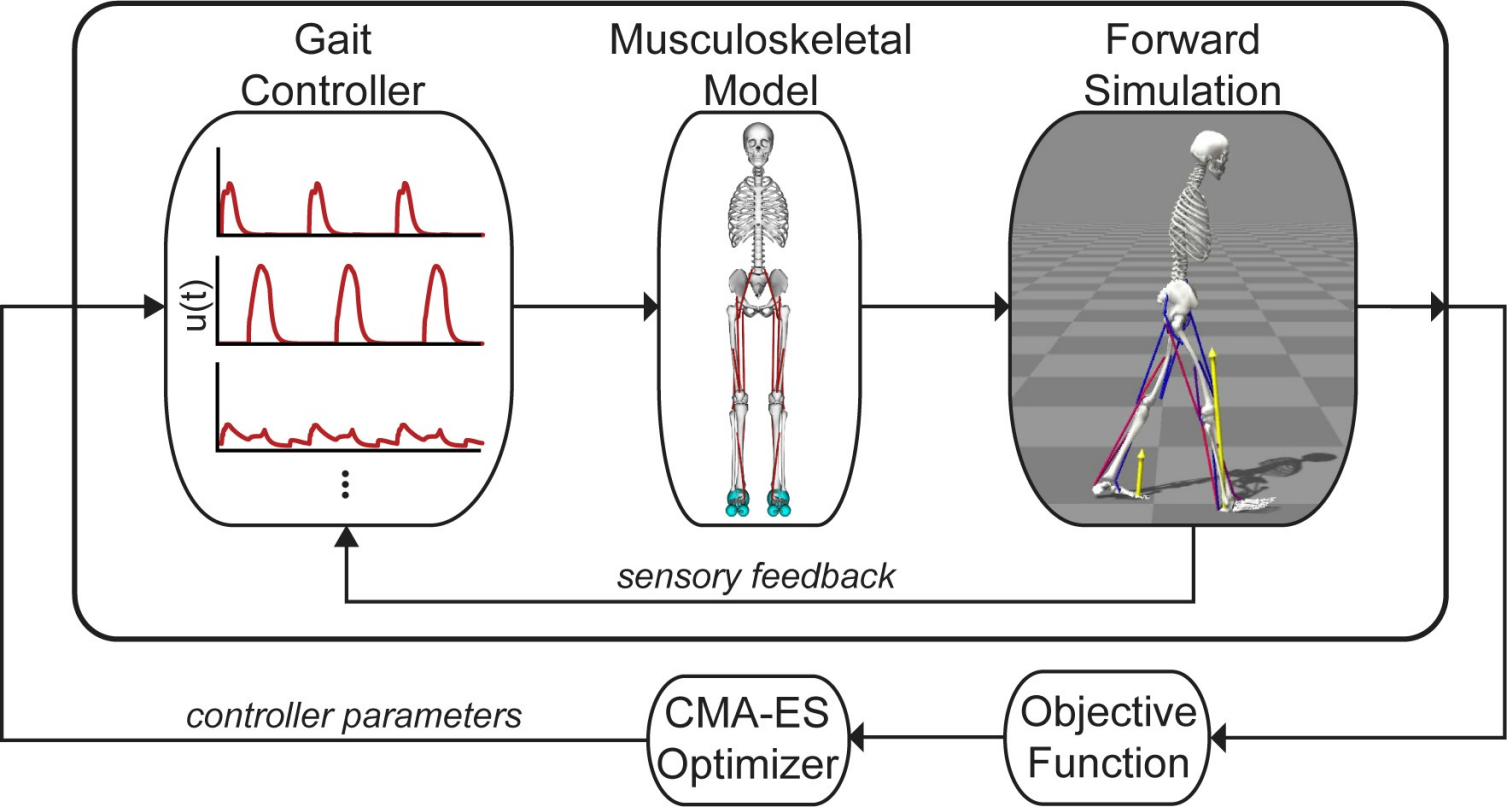
Single shooting framework for dynamic optimization. We trained a planar, musculoskeletal model actuated by 18 Hill-type musculotendon actuators to walk by optimizing the parameters of a gait controller based on an objective function that sought to minimize metabolic cost, avoid falling and injury, and stabilize the head. The gait controller computed muscle excitations, *u(t)*, for a musculoskeletal model to generate a forward simulation. Sensory feedback, based on the model’s muscle and joint states, was used in a feedback loop with the gait controller. The objective function quantified the performance of each simulation, and a Covariance Matrix Adaptation Evolutionary Strategy (CMA-ES) optimization method updated the values of the variables in the optimization problem.

Our optimization framework used a single shooting method to solve the dynamic optimization problem of generating a simulation of gait (Fig 1). We implemented our model in OpenSim 3.3 [31,32] and used an optimization and control framework (SCONE) to implement the gait controller, perform the simulation using OpenSim as the plant, and optimize the parameters of our problem.

### Musculoskeletal model

**Fig 2:**
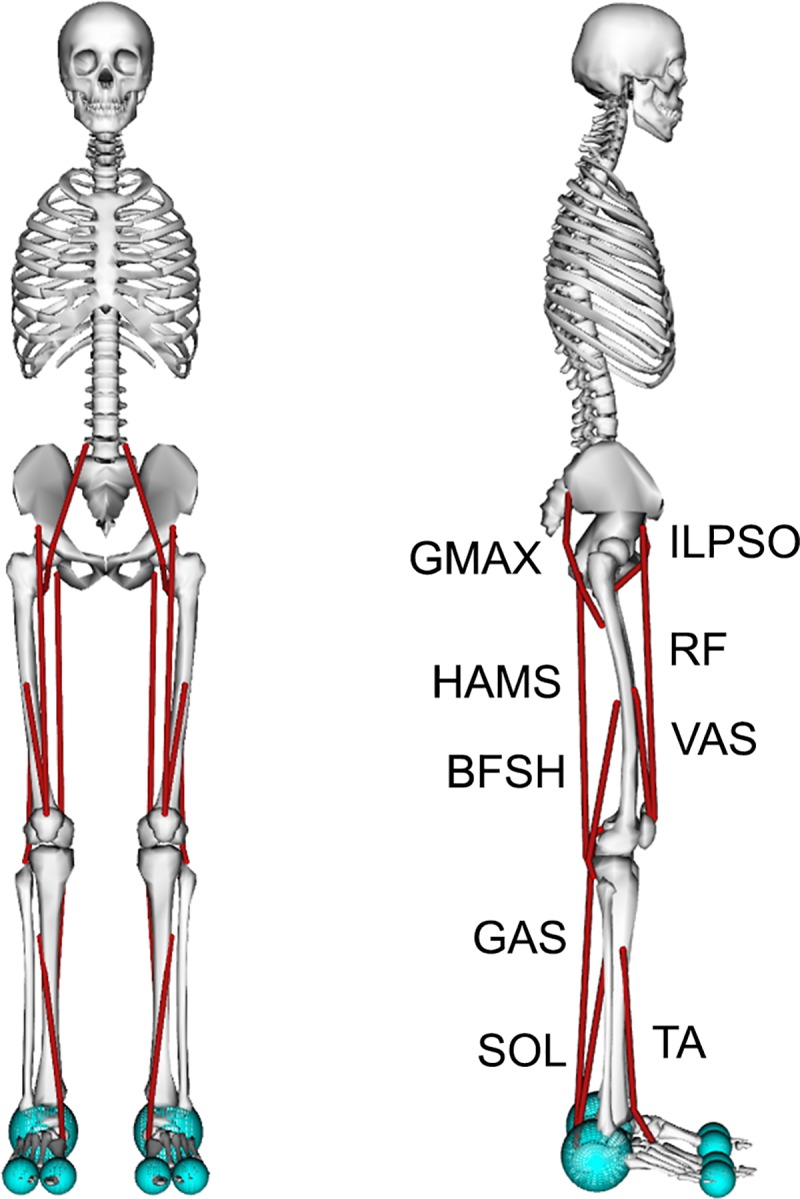
A planar musculoskeletal model for walking. The musculoskeletal model had nine degrees of freedom (dof). A 3-dof planar joint between the pelvis and ground was the base joint. Each hip and ankle was a 1-dof pin joint, and each knee was a 1-dof coupled joint. The model’s 18 musclulotendon actuators (red lines) represented the 9 major uniarticular and biarticular muscle groups per leg that drive sagittal plane motion: gluteus maximus (GMAX), biarticular hamstrings (HAMS), iliopsoas (ILPSO), rectus femoris (RF), vasti (VAS), biceps femoris short head (BFSH), gastrocnemius (GAS), soleus (SOL), and tibialis anterior (TA). A compliant contact model was used to generate forces between the spheres at the heel and toes of the feet and the ground plane.

The musculoskeletal model (Fig 2) was a planar model based on a previous model that represented an adult with a height of approximately 1.8 m and a mass of 75.16 kg and was used to study the lower limb [33]. The musculoskeletal model had nine degrees of freedom (dof). Planarity was achieved by a 3-dof planar joint between the pelvis and ground. A 1-dof pin joint represented each hip and ankle, and a 1-dof joint with coupled rotation and translation represented each knee. The lumbar joint was locked at 5° of flexion based on previous walking data [2].

The model was actuated using 18 Hill-type muscle-tendon units [34] with 9 per leg. Muscles with similar functions in the sagittal plane were combined into single muscle-tendon units with combined peak isometric forces to represent 9 major muscle groups per leg: gluteus maximus (GMAX), biarticular hamstrings (HAMS), iliopsoas (ILPSO), rectus femoris (RF), vasti (VAS), biceps femoris short head (BFSH), gastrocnemius (GAS), soleus (SOL), and tibialis anterior (TA). Peak isometric forces were based on a previous musculoskeletal model [35], whose muscle volumes were based on young, healthy subjects [36]. The tendon strain at peak isometric force was 4.9% [35,37] for all muscles except for the plantarflexors, whose values were set to 10% [38]. To better match passive muscle forces during walking [39], we adjusted the passive force-length curves of the HAMS, VAS, and RF to decrease their passive forces (S1 Table). The tendon slack length of each muscle was calculated based on experimental data [40], as done previously in other musculoskeletal models [35,41]. We set maximum muscle fiber contraction velocity to 15 optimal fiber lengths per second ($l_{o}^{m}/s$) as used in previous studies [38,42], because models that represent muscle paths as a single line tend to overestimate length changes [43,44]. All muscle-tendon parameters are summarized in S1 Table.

Ligaments were modeled as nonlinear, rotational springs that generated torques when a joint was hyperflexed or hyperextended. In our model, ligaments generated torques when the hip was flexed beyond 120° or extended beyond 30°, the knee was flexed beyond 140° or extended beyond 0°, and the ankle was dorsiflexed beyond 20° or plantarflexed beyond 40°.

A compliant contact model [45,46] was used to generate forces between the feet and the ground. Each foot had three contact spheres: one sphere with a radius of 5 cm represented the heel, and two spheres, each with a radius of 2.5 cm, represented the toes. All contact spheres had the following parameters: plane strain modulus of 500,000 N/m^2^, dissipation coefficient of 1.0 s/m, static and dynamic friction coefficients of 0.8, viscous friction coefficient of 0, and transition velocity of 0.1 m/s.

We modeled mild, moderate, and severe weakness by reducing a muscle’s maximum isometric force ($F_{o}^{m}$) to 25%, 12.5%, and 6.25%, respectively, of its original value. The resulting values for $F_{o}^{m}$ were similar to those used in a previous study that computed the minimum plantarflexor muscle strength needed to walk either normally or in a crouch [19]. We modeled mild, moderate, and severe contracture by reducing a muscle’s optimal fiber length ($l_{o}^{m}$) to 85%, 70%, and 55%, respectively, of its original value. The severe case was based on experimental data [47] and previously used as a model of contracture [48]. We separately applied each of these 6 deficits to the soleus only (SOL), gastrocnemius only (GAS), or both plantarflexors (PF), to yield 18 total deficit cases.

### Gait controller

The gait controller was based on previous reflex-based controllers for human locomotion [21,22,24] and used a combination of a high-level state machine and low-level control laws to calculate muscle excitations. Our controller had 5 high-level states that represented key points in the gait cycle for each leg: early stance (ES), mid-stance (MS), pre-swing (PS), swing (S), and landing preparation (LP). Of the 5 transitions between these high-level states, transitions between 4 of the states were controlled by 4 thresholds that were free parameters in the optimization: 1) ES to MS: horizontal distance between the ipsilateral foot and pelvis was lower than a threshold; 2) PS to S: magnitude of ground reaction force (GRF) on the ipsilateral foot was lower than a threshold; 3) S to LP: horizontal distance between the ipsilateral foot and pelvis was greater than a threshold; and 4) LP to ES: magnitude of GRF on ipsilateral foot was greater than a threshold. The fifth transition, MS to PS, was not controlled by a free parameter and occurred when the contralateral leg entered the ES state.

**Fig 3:**
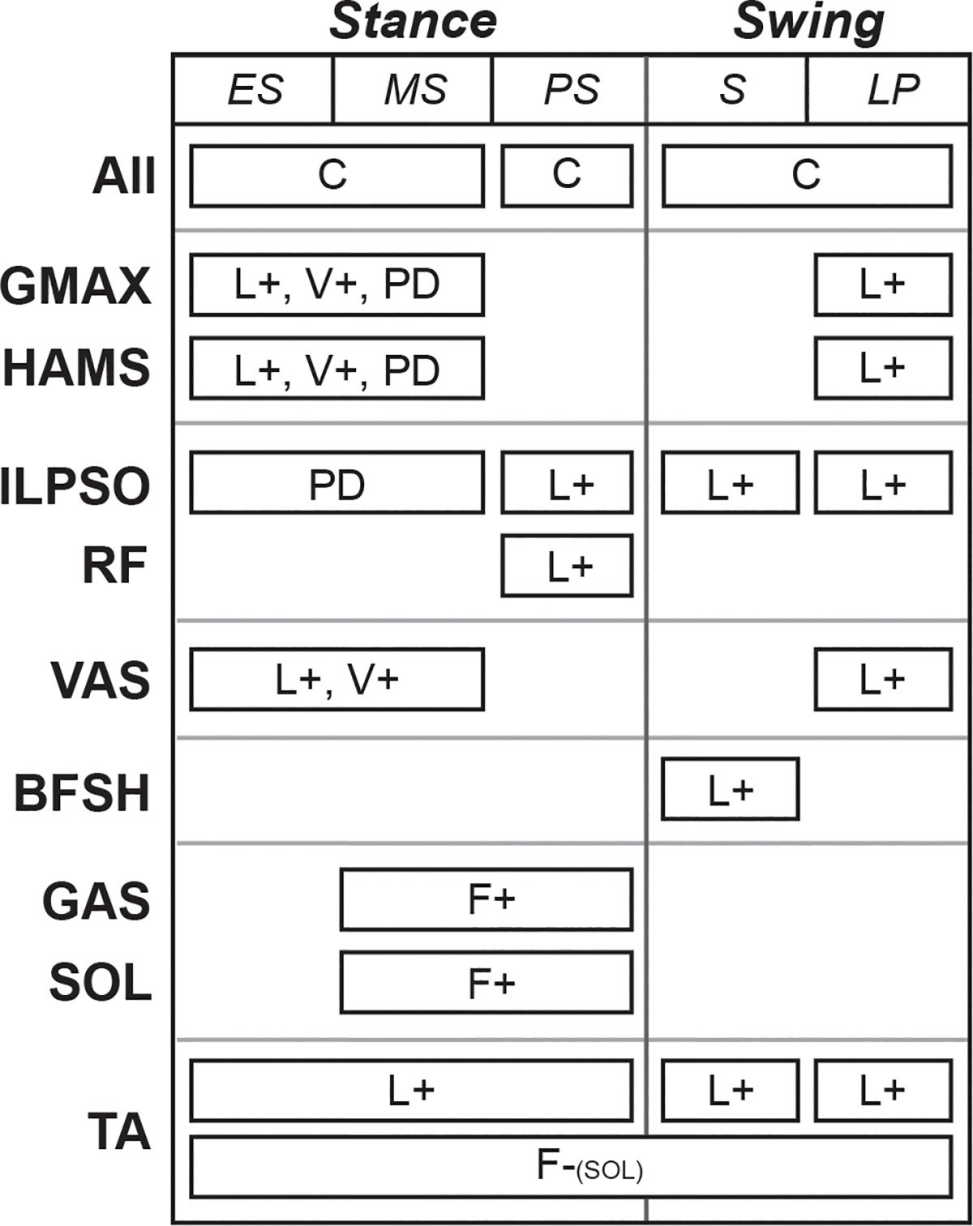
The gait controller used a combination of a state machine and low-level control laws to determine excitations. The state machine had three states in stance (i.e., early stance (ES), mid-stance (MS), and pre-swing (PS)) and two states in swing (i.e., swing (S) and landing preparation (LP)), and it determined when low-level control laws were active. Low-level control laws included constant signals (C), feedback terms based on muscle length (L), muscle velocity (V), and muscle force (F), and proportional-derivative control (PD) based on the pelvis tilt angle. Positive and negative feedback are denoted by (+) and (-), respectively. All feedback laws based on muscle states acted upon the same muscle, except for a negative force feedback from the soleus to the tibialis anterior.

Low-level control laws were active or inactive depending on the high-level controller (Fig 3). There were five types of control laws to generate muscle excitations (*u*). One control law, constant (C) control, did not depend on feedback from muscle or joint states. Three control laws depended on muscle feedback: length feedback (L), velocity feedback (V), and force feedback (F). The last control law (PD) depended on feedback from joint positions and velocities, similar to proportional-derivative control. All muscle-based feedback laws were positive feedback laws (i.e., L+, V+, F+) onto the same muscle, except for a negative force feedback law (F-) from the soleus to the tibialis anterior. PD control was only used to control the pelvis tilt angle (*θ*) and velocity ($\dot{\theta }$) with respect to ground using the muscles crossing the hip joint (i.e., ILPSO, GMAX, and HAMS). Excitations were calculated over time (*t*) by the following equations:

$$u_{C}=K_{C}$$ (1)

$$u_{L+}=max(0,K_{L+}[l(t-t_{D})-l_{o}])$$ (2)

$$u_{V+}=max(0,K_{V+}[v(t-t_{D})])$$ (3)

$$u_{F\pm }={\pm K}_{F\pm }F(t-t_{D})$$ (4)

$$u_{PD}=K_{p}[\theta (t-t_{D})-\theta_{o}]+K_{v}[\dot{\theta }(t-t_{D})]$$ (5)

States calculated from the model were muscle length (*l*), muscle velocity (*v*), muscle force (*F*), and pelvis tilt orientation (*θ*) and velocity ($\dot{\theta }$). In total, there were 70 free parameters of the optimization, including the controller gains (*K*_*C*_, *K*_*L+*_, *K*_*V+*_, *K*_*F±*_, *K*_*p*_, and *K*_*v*_), and offsets for muscle length feedback (*l*_*o*_) and proportional feedback of *θ* (*θ*_*o*_). For all positive feedback and PD control laws, the parameter for time delay, *t*_*D*_, was set for each muscle depending on the most proximal joint over which the muscle crosses based on values from previous simulation work using reflex-based controls [21–24]: *t*_*D*_ was 5 ms for the hip, 10 ms for the knee, and 20 ms for the ankle. For the F- law from the soleus to the tibialis anterior, *t*_*D*_ was 40 ms. When considered with a first-order activation time constant of 10 ms, this delay better represented the short-latency TA suppression (56 to 74 ms) observed in experiments [49]. Overall, however, the delays used were still shorter than those measured experimentally [50]. Muscle activations were calculated from the muscle excitations using a first-order dynamic model, with activation and deactivation time constants of 10 ms and 40 ms, respectively [34].

### Simulation and optimization framework

OpenSim was used to form and integrate the model’s equations of motion [31,32]. The positions and velocities of each of the nine degrees of freedom of the model were initialized by first setting the pelvis’s horizontal position to 0 m. Then, we set initial angular positions and velocities, and the velocity of the pelvis according to free parameters in the optimization. Finally, the vertical position of the pelvis was set such that the GRF was equal to half of the model’s body weight. Thus, of the 18 initial skeletal states, 16 states were set by free parameter optimization. One issue with optimizing velocities was that this may have added extra energy into the system because, for example, the optimizer could have chosen a high initial pelvis velocity value. This likely did not affect our results as we found step-to-step variability to be small, and we only analyzed kinematics and kinetics after the first two steps taken. Muscle states were set by equilibrating the force between the muscle and tendon at an activation based on the initial excitations calculated by the gait controller. Integration continued until the desired simulation time was reached or until the model fell, which we defined as the model’s center of mass (COM) height falling below 0.8 times the height of the COM at the start of the simulation.

To evaluate a set of design parameters, we defined an objective function, *J*, which we sought to minimize. The objective, *J*, quantified high-level tasks of walking:

$$J=w_{cot}J_{cot}+w_{spd}J_{spd}+w_{inj}J_{inj}+w_{head}J{}_{head}$$ (6)

The goal was to minimize the gross cost of transport (*J*_cot_) while maintaining a prescribed average speed over each step within a range, avoiding falling (*J*_spd_), avoiding ligament injury (*J*_inj_), and stabilizing the head (*J*_head_). To balance competing objectives, the weights were manually adjusted to the following: *w*_cot_ = 1 kg·m/J, *w*_spd_ = 10,000 s^-1^, *w*_inj_ = 0.1 (N·m)^-2^s^-1^, and *w*_head_ = 0.25 s^3^/m^2^. These weights prioritized finding solutions in which the model avoided falling before finding ones that minimized cost of transport, injury, and accelerations at the head. For optimized solutions, the contribution from the *J*_head_ term was larger than those from the *J*_spd_ and *J*_inj_ terms, and thus our results were most sensitive to our choice of *w*_head_. Because of this, our choice for *w*_head_ represents a trade-off between head stability, especially during early stance, and cost of transport.

*J*_cot_ penalized simulations in which the model walked in a less metabolically efficient manner [51] and was calculated by summing the basal and per-muscle metabolic rates. To calculate metabolic rate, we used the implementation of Uchida et al. [52] based on a previous muscle metabolic model by Umberger et al. [53] to estimate the gross metabolic rate ($\dot{E}$) over the duration of the simulation (*t*_end_), and normalized by the mass of the model (*m*) and distance travelled (*d*):

$$J_{cot}=\frac{\int_{0}^{t_{end}}\dot{E}dt}{md}$$ (7)

*J*_spd_ penalized simulations in which the model fell before the desired simulation end time (*t*_des_) and had step speeds outside of a prescribed range. *J*_spd_ was calculated using the following equations:

$$J_{spd}=1\;s-\sum_{s}\frac{{t_{s}v}_{s,pen}}{v_{min}}$$ (8)

$$v_{s,pen}=\left\{ \begin{array}{r} 0,\;t_{end}=t_{fall}\\ \max\left[0,\;1\;m/s-\Psi (v_{s},v_{min},v_{max})\right],\;t_{end}=t_{des} \end{array}\right.$$ (9)

$$\Psi (\varphi ,\varphi_{min},\varphi_{max})=\left\{ \begin{array}{l} \;0,\;\varphi_{min}<\varphi <\varphi_{max}\\ \varphi_{min}-\varphi ,\;\varphi <\varphi_{min}\\ {\varphi -\varphi }_{max},\;\varphi >\varphi_{max} \end{array}\right.$$ (10)

where *s* denotes a step, *t*_*s*_ is the duration of a step, *v*_*s*,pen_ is a penalty for a given step (ranging between 0 and 1, where 1 is no penalty), *v*_*s*_ is the step speed, *v*_min_ and *v*_*max*_ define the minimum and maximum speeds allowed without a penalty, *t*_fall_ is the time at which a fall occurred, and Ψ applies a linear penalty if *v*_*s*_ is not between *v*_min_ and *v*_max_. If all steps were within the speed range, *J*_spd_ was 0, and if the model immediately fell, *J*_spd_ was 1. For any given step in which the speed was outside of the range, the step was penalized more heavily (i.e., *v*_*s*,pen_ was closer to 0) in a linear fashion the further the speed was out of the range, and its final contribution to *J*_spd_ was scaled by *t*_s_. For simulations with a prescribed speed, the speed range was ±0.05 m/s of the prescribed speed. For example, to prescribe a speed of 1.25 m/s, *v*_min_ = 1.20 m/s and *v*_max_ = 1.30 m/s. For simulations without a prescribed speed, *v*_min_ was 0.75 m/s, and *v*_max_ was arbitrarily large.

*J*_inj_ penalized simulations in which the model engaged the ligaments and was calculated by integrating the sum of joint torques (*T*_*j*_) squared generated by the model’s ligaments:

$$J_{inj}=\int_{0}^{t_{end}}\sum_{j}T_{j}^{2}dt$$ (11)

Since head stability is commonly regarded as an important task during gait [54], *J*_head_ penalized simulations in which the model had excessively large accelerations and was calculated by the following equation and Eq 10:

$$J_{head}=\int_{0}^{t_{end}}\left[{\Psi (a_{x},a_{x,min},a_{x,max})}^{2}+{\Psi (a_{y},a_{y,min},a_{y,max})}^{2}\right]dt$$ (12)

where *a*_*x*_ and *a*_*y*_ are the horizontal and vertical accelerations at a point located near the center of the head, and *a*_*x*_,_min_, *a*_*x*_,_max_, *a*_*y*_,_min_, and *a*_*y*_,_max_ determine the bounds between which no penalty is applied if *a*_*x*_ and *a*_*y*_ are within the bounds. We set our acceleration bounds as *a*_*x*_,_min_ = -0.25*g*, a_*x*,max_ = 0.25*g*, a_*y*,min_ = -0.50*g*, and *a*_*y*,max_ = 0.50*g*, which we approximated from experimental data of human walking [55] and where *g* = 9.80665 m/s^2^.

To solve our optimization problem, we used an evolutionary strategy called Covariance Matrix Adaptation Evolutionary Strategy (CMA-ES) with a rank-μ update [56]. In total, there were 90 design variables: 74 for the gains, offsets, and transition parameters in the gait controller, and 16 for the parameters that set the model’s initial state. Refer to S2 Table for the initial standard deviations used for each parameter. We set the CMA-ES parameters such that the population size of each generation (*λ*) was 16 and the update step for each generation used only the 8 best solutions (*μ*). Our optimizations were set to run for a given number of CMA-ES iterations; this number was set to be large enough for the objective function to stop improving significantly at the end of the optimization. The number of iterations differed based on the desired simulation length of time and is detailed in the next section. Because these types of problems tend to have noisy, non-convex search spaces, and CMA-ES is a stochastic algorithm, we ran a set of multiple parallel optimizations with the same initial guess and used the best solution from the set to seed the next set of optimizations as has been done previously [24,57]. We chose to stop when the best solution from the set of parallel optimizations had an objective function value that was no better than 5% of the one from the previous set of optimizations, as we observed optimized solutions that were within 5% of each other were qualitatively similar.

### Generating simulations of gait

To generate simulations of gait with a prescribed speed, we used 10 second simulations (*t*_des_ = 10 s) and a set of 20 parallel optimizations, each with a maximum of 3000 generations for CMA-ES. There were 7 simulations that were generated at prescribed speeds between 0.50 m/s and 2.00 m/s at an interval of 0.25 m/s. We first trained the model at the middle speed, 1.25 m/s, and then used those results to seed the neighboring speeds, continuing until we reached the lowest and highest speeds. For example, the solution for our 1.25 m/s case was used to seed the 1.00 m/s case, which was then used to seed the 0.75 m/s case.

To generate simulations of gait with a self-selected speed, we used longer, 30 second simulations (*t*_des_ = 30 s), as we found that solutions with a shorter desired simulation time would systematically fall forward at the end without a tight bound on step speed. We used a set of 10 parallel optimizations, each with a maximum of 1500 generations for CMA-ES. We generated 3 simulations of unimpaired gait at a self-selected speed, seeded from solutions of the slowest (0.50 m/s), middle (1.25 m/s), and fastest (2.00 m/s) cases from above. We also generated 18 simulations of impaired gait, starting with the unimpaired solution and seeding the next more severe case. For example, the solution from the unimpaired case was used to seed the mild SOL weakness case, which was used to seed the moderate case, which was finally used to seed the severe case.

To further test if our method could generate stable gait at a self-selected speed with our unimpaired model, we simulated the self-selected gait solution seeded from the middle case with a target simulation time of 1 hour (*t*_des_ = 3600 s).

### Computational resources

Each optimization was run on a node of a computational cluster with two Xeon E5-2650v2 processors, containing 16 cores total, at 2.60 GHz. Each optimization problem took between 13 and 20 hours to complete. To perform parallel optimizations, we used the same number of parallel nodes, so the time to complete a set of optimizations was similar to the time to complete a single optimization.

## Results

We first compare our simulations of unimpaired walking to experimental kinematic, kinetic, metabolic, and muscle activity data. We then discuss the kinematic and kinetic adaptations caused by introducing plantarflexor weakness or contracture into our musculoskeletal model. Video highlights of our results are provided in S1 Video, and videos of all simulations discussed here are provided in S2 Video.

### Validating the model’s gait over a range of speeds

We validated the ability of our model and optimization framework to capture trends in kinematics, kinetics, and spatiotemporal measures when walking at different speeds by generating seven simulations of gait at prescribed speeds between 0.50 m/s and 2.00 m/s, at intervals of 0.25 m/s. All simulations found a steady gait pattern at a speed within 0.05 m/s of the speed prescribed by the optimization’s objective function (S2 Video). Individual comparisons of each speed to experimental data from Schwartz et al. [58] are provided in S1 Fig.

**Fig 4:**
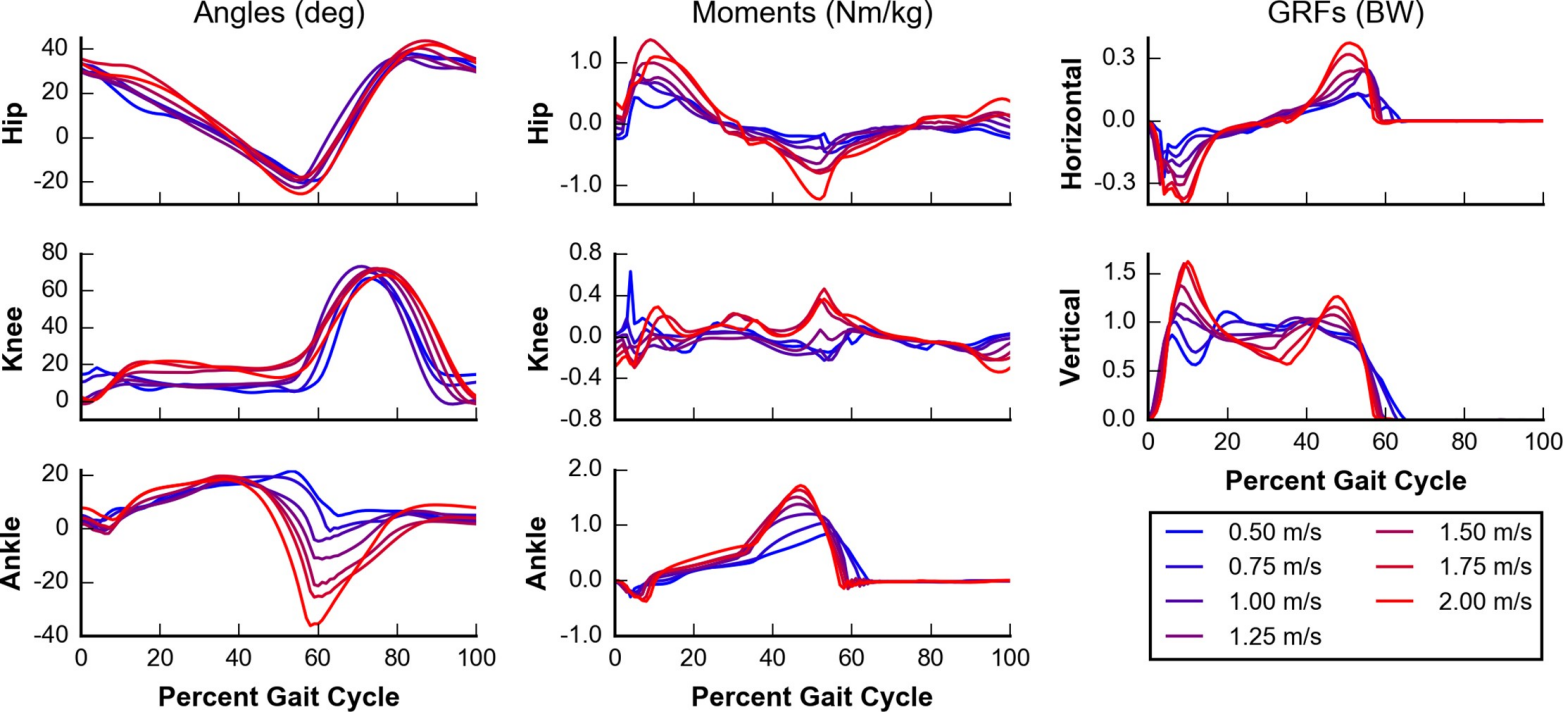
Kinematics and kinetics of simulated walking over a range of speeds. Seven prescribed speeds between 0.50 m/s (blue) and 2.00 m/s (red) at intervals of 0.25 m/s were analyzed. Joint angles (left column) and joint moments (middle column) are plotted for the hip (top row), knee (middle row), and ankle (bottom row). Positive joint angles indicate flexion, and positive joint moments indicate extension. Ground reaction forces (GRFs) (right column) are shown for both horizontal (top row) and vertical (middle row) directions. With exceptions at the knee joint, we observed trends that occur in the experimental data of Schwartz et al. [58], including greater joint angle ranges, joint moments, and ground reaction forces at higher speeds.

Simulated kinematic and kinetic adaptations, including those in joint angles, joint moments, and GRFs, matched trends observed in experimental data [58] with increasing speeds (Fig 4). Hip and ankle joint angle excursions increased with increasing speeds. Kinetic data, such as peak flexion and extension moments about the hip, knee, and ankle, and the peak horizontal and vertical GRFs, also increased with increasing speeds. Some experimental trends, however, were not captured in our simulations. The knee joint angle range and peak flexion during swing did not increase at higher speeds. At the slowest prescribed speeds (0.50 m/s and 0.75 m/s), the GRFs show low-frequency oscillations not found in experimental data. This was likely due to our choice for contact parameters, which yielded a softer contact than previous work [23]. While this choice likely contributed to resonance, it also sped simulations up by roughly 2- to 3-fold. We note one aberrant point for the simulation at the slowest speed (0.50 m/s) in the knee joint moment plot that shows a spike of knee extension in early stance, but it does not appear to affect other kinematic or kinetic measures. Overall, while these results show that our simulations capture many salient kinematic and kinetic trends, we must be cautious in interpreting GRF results at speeds at or below 0.75 m/s.

**Fig 5:**
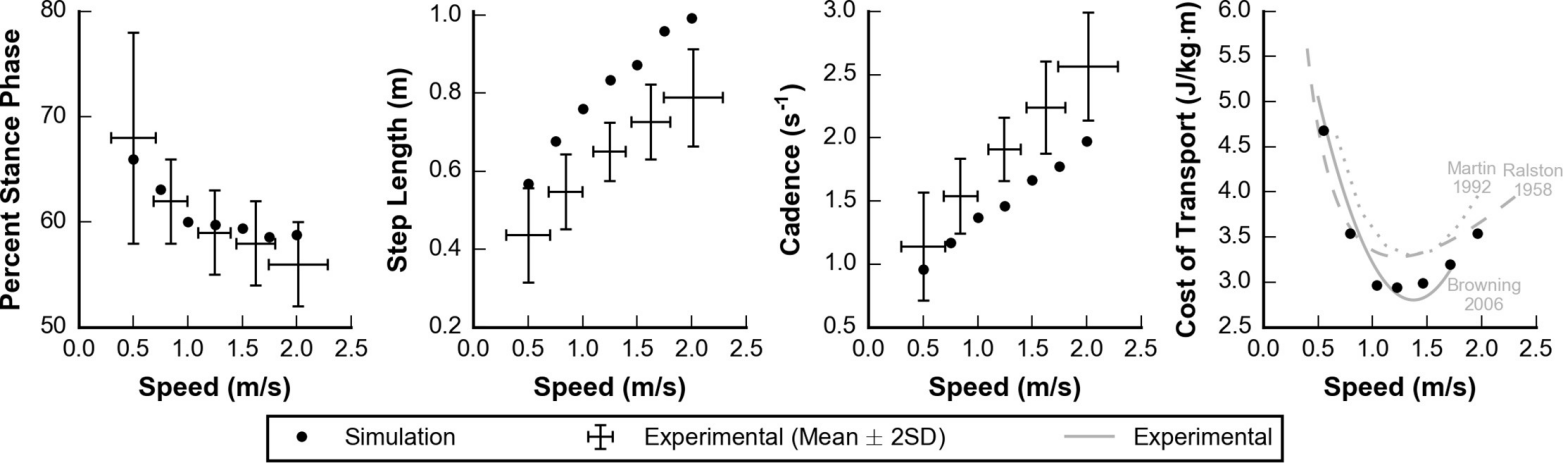
Spatiotemporal and metabolic parameters over a range of speeds. Simulated (dots) and experimental (lines) data are plotted for percent stance phase (left), step length (middle left), cadence (middle right), and cost of transport (right). Experimental data for the left three panels represent the mean ± 2 standard deviations from Schwartz et al. [58]. In the right panel, the magnitude and characteristic shape of the cost of transport bowl were similar between the simulated data and three experimental data sets from Ralston, Martin et al., and Browning et al. [59–61].

Percent stance phase (i.e., the percent of time each foot is in stance during a gait cycle) decreased, and step length and cadence increased with increasing speeds as observed experimentally by Schwartz et al. [58] (Fig 5). Percent stance phase was within 2 standard deviations (SD) of experimental data at all speeds. We note that because the experimental data contained subjects between the ages of 4 and 17, the SDs may be larger than data containing adults alone. Although the trends of step length and cadence in our simulations followed experimental data, the simulations generated gaits with a longer step length and a slower cadence than observed experimentally. Our simulations were also able to reproduce experimentally observed trends in metabolic cost of transport, as measured by Ralston, Martin et al., and Browning et al. [59–61], over the range of speeds we tested (Fig 5, right panel). In particular, a greater increase in cost of transport was observed when walking slower than optimal as compared to faster than optimal.

### Validating the model’s gait at self-selected speed

We tested the ability of our model and optimization framework to generate a realistic gait at self-selected speed by generating simulations that did not have a prescribed speed within the optimization’s objective function. Instead, we imposed a minimum speed of 0.75 m/s, which helped guide the optimizer to better solutions but was sufficiently low that it did not affect the final optimized solutions. We generated 3 simulations by initializing the optimization framework with the optimized parameters for 3 of the prescribed speeds from the previous section: slowest (0.50 m/s), middle (1.25 m/s), and fastest (2.00 m/s) speeds. The optimization yielded 3 simulations with similar speeds of 1.23 m/s, 1.21 m/s, and 1.29 m/s, respectively. Each speed was within 2 SDs of the expected self-selected speed (1.25 ± 0.15 m/s) for an individual of our model’s leg length [58]. This suggests that our optimization framework can robustly find solutions and is not sensitive to initial guess. For simplicity, only the simulation initialized with the middle speed is described in the remainder of this section, and all three simulation results are provided in S2 Fig.

**Fig 6:**
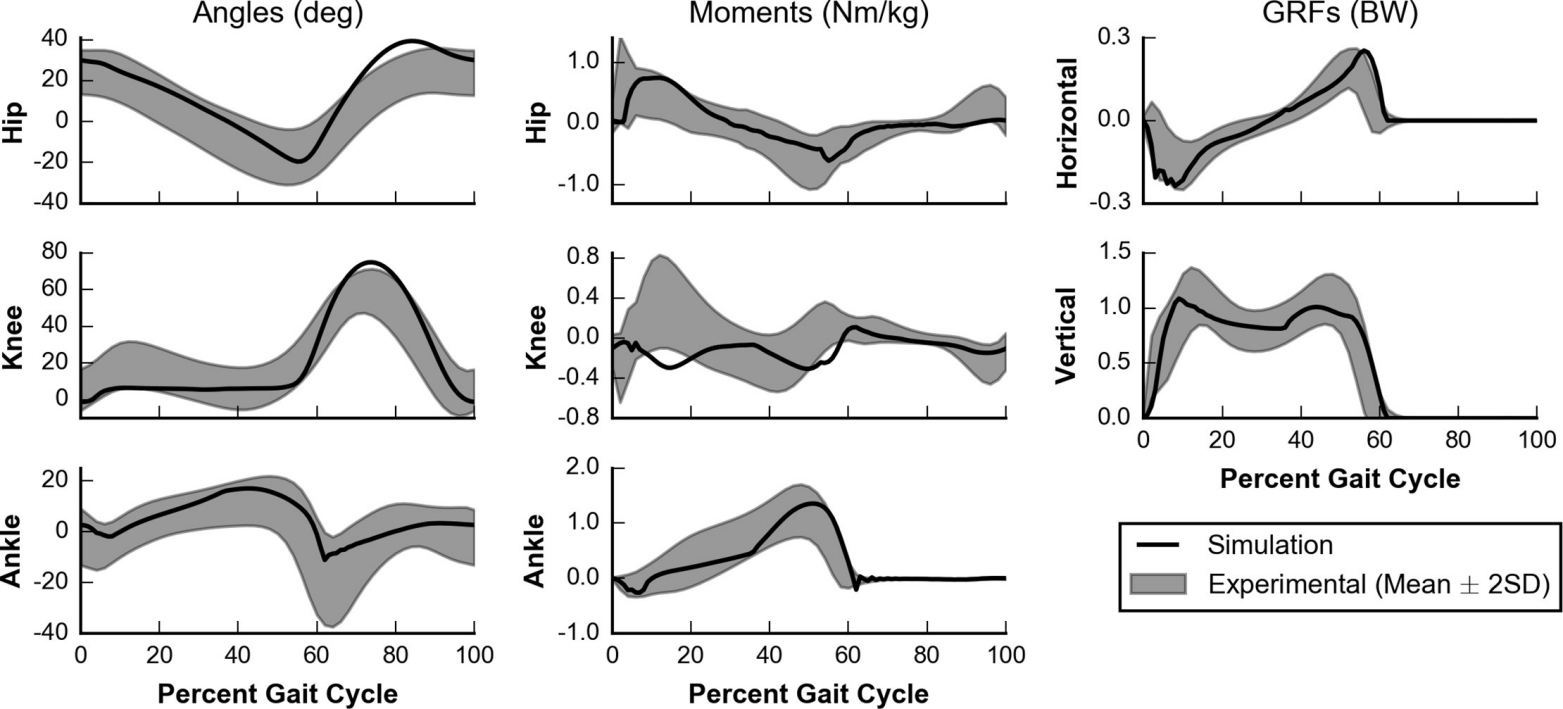
Kinematics and kinetics of gait at self-selected speed. Simulated (black line) kinematics and kinetics are compared to experimental data (gray area) collected by Schwartz et al. [58]. Joint angles (left column) and joint moments (middle column) are plotted for the hip (top row), knee (middle row), and ankle (bottom row). Ground reaction forces (right column) are shown for both horizontal (top row) and vertical (middle row) directions. Positive angles indicate flexion, and positive moments indicate extension. Note that the experimental data for hip flexion angle was shifted by 11.6° to account for the difference in pelvis orientation definitions between the experimental data set and the musculoskeletal model.

Our simulated kinematic and kinetic trajectories, including joint angles, joint moments, and GRFs, were similar both in value and shape to experimental data reported by Schwartz et al. [58] for gait at self-selected speed (Fig 6). For the majority of the gait cycle, simulated trajectories were within 2 SDs of experimental data [58]. For each of the trajectories, the root-mean-squared error (RMSE) between simulated and experimental data was no more than 1.55 SD, and for each trajectory except the knee extension moment, the normalized cross-correlation (NCC) [62], which measures shape similarity between the simulated and experimental data sets, was at least 0.82 (Table 1).

**Table 1:** Similarity metrics between simulated and experimental data for self-selected gait.

	Angles			Moments			GRFs (stance only)	
	Hip	Knee	Ankle	Hip	Knee	Ankle	Horizontal	Vertical
**Root-mean-squared error (SD)**	1.55	1.48	0.82	1.08	1.46	1.17	1.04	0.78
**Normalized cross-correlation**	0.95	0.97	0.82	0.83	-0.04	0.95	0.95	0.99

Root-mean-squared error compares simulation mean trajectories to experimental mean and standard deviation, reported in units of standard deviation (SD). Given the experimental mean and standard deviation over the gait cycle, we compute a Z-score for the simulation data at every 1% of the gait cycle. Then, we take the square root of the sum of squares of all the Z-scores. Normalized cross-correlation compares mean trajectories between simulation and experimental data [58].

The three trajectories with the highest RMSEs, hip joint angle (1.55 SD), knee joint angle (1.48 SD), and knee joint moment (1.46 SD), each contained times when the simulation deviated by more than 2 SDs from the experimental mean. The hip and knee joint angles showed excessive flexion during the swing phase. These were compensations likely due to the simplifications in the model, as degrees of freedom that contributed to foot clearance, such as pelvic list and hip abduction, were not included in the model.

All NCCs were above 0.9 except those for comparisons between simulated and experimental knee joint angle, hip joint moment, and knee joint moment trajectories. Despite the larger RMSEs, the higher NCCs of the simulated hip (0.95) and knee (0.97) joint angles to their corresponding experimental trajectories indicate the shape of the simulated and experimental trajectories matched well. The NCC for the knee joint angle trajectory of 0.82 was lower than most other trajectories, likely due to the lack of knee flexion during early stance in our simulations. The lower NCC for the hip joint moment trajectory of 0.83 is likely due to a lack of hip extension moment in late swing and just after initial contact. The simulated and experimental knee extension moments did not have a similar shape, with a NCC of -0.04. However, there were large standard deviations in the experimental data during this part of the gait cycle, which is not considered when computing NCC. Differences during early stance explain this discrepancy in knee joint moment trajectories, as the model generated a knee flexion moment when experimental data exhibits a knee extension moment. The model took long steps and landed with an extended knee, causing the GRF to generate a knee extension moment, rather than a knee flexion moment in early stance. Thus, the muscles had to generate a knee flexion moment, rather than a knee extension moment, to prevent hyperextension.

**Fig 7:**
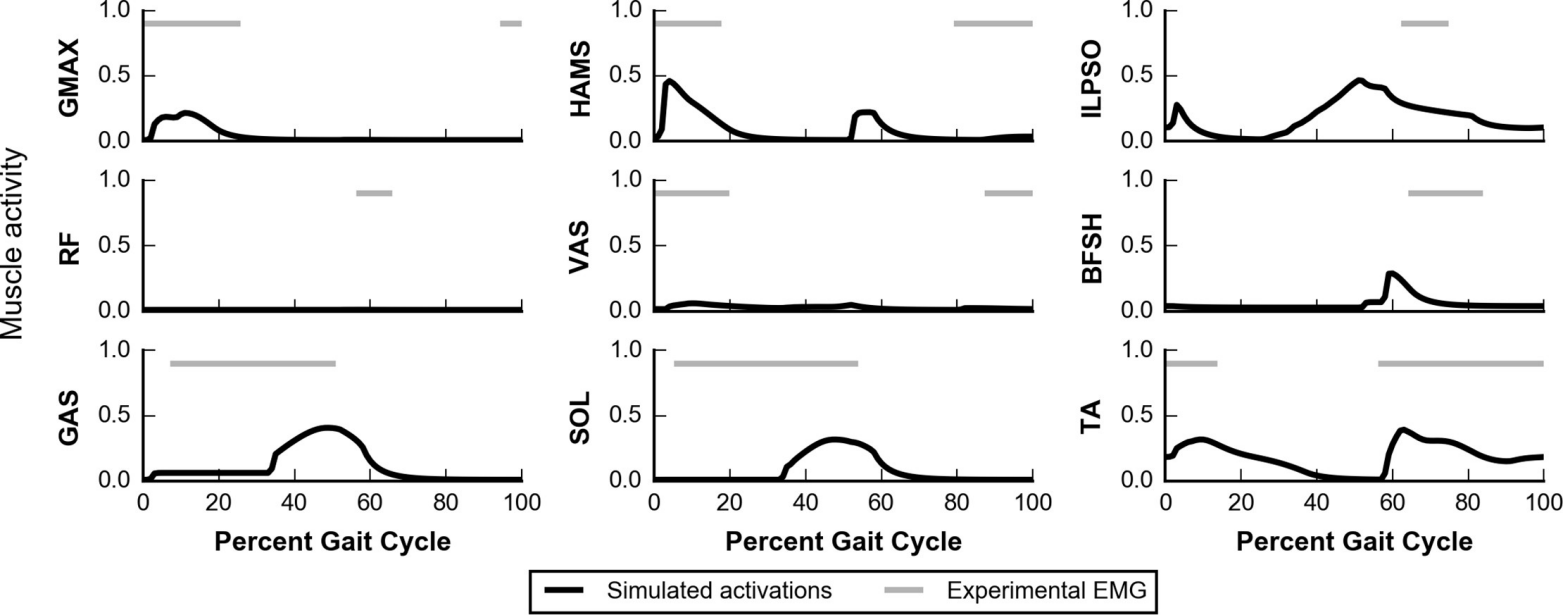
Muscle activity during gait at self-selected speed. Simulated muscle activations (black) are compared to on-off timings estimated from experimental electromyograms (EMG) reported by Perry and Burnfield (gray) [63]. Because the muscles in the model represent groups of muscles, we show on-off timing of multiple muscles for some comparisons (e.g., for HAMS, experimental data represent the long head of the biceps femoris, semitendinosus, and semimembranosus). S3 Table contains more details about these comparisons.

We compared trajectories of simulated muscle activations with on-off timings estimated from experimental electromyograms (EMG) as reported by Perry and Burnfield [63] (Fig 7). The simulated muscle activity captured many salient features observed in experiments. During early stance, the hip extensors, GMAX and HAMS, were active for weight acceptance. During the latter half of the stance phase, the plantarflexors GAS and SOL were used for propulsion. The dorsiflexor TA was active both throughout swing and during early stance. There were some differences between our simulated data and experimental EMG. VAS activity was lower during early stance in our simulations than in experimental data. As previously discussed, our simulation did not generate a knee extension moment in early stance, possibly due to the model taking long steps and landing with an extended knee, which could have led to the model avoiding activation of the VAS. The onset times for GAS and SOL were later in our simulation than observed in experiments. To generate a hip flexion moment during swing, our model used ILPSO, which was active for more time than experimental iliacus data. This likely occurred because the model lacks other muscles that can generate a hip flexion moment, such as adductor longus and sartorius. RF was not used for hip flexion in our simulation; however, RF activity during pre-swing has been reported to be inconsistent between subjects during normal walking [63], with more consistent RF activity at higher speeds [64]. To generate a knee flexion moment during pre-swing, HAMS were activated in our simulation, which was only seen in a minority of experimental subjects by Perry and Burnfield [63]. BFSH activity occurred earlier in the gait cycle in our simulation than in experiments. Just prior to initial contact, experimental subjects show activity in the hip extensors GMAX and HAMS; however, our simulation only showed minimal HAMS activity, explaining the lack of hip extension moment during this phase.

We also tested if our self-selected gait solution was stable to small numerical perturbations, such as those arising from integration differences. Using the same solution that was trained with a target time of 30 s, we set a target time of 1 hour and found that our model could generate a stable walk for at least 1 hour. This indicated that, for an unimpaired model, our method could find solutions that were stable for much longer than the target time during optimization of 30 s.

### Simulating walking with plantarflexor weakness or contracture

The results of the previous two sections gave us confidence that our model and optimization framework could generate realistic gaits over a wide range of speeds and could self-select a realistic gait and speed. We then used the framework to study how gait would adapt to deficits in the plantarflexors by adjusting the model’s parameters and using the same optimization framework.

**Fig 8:**
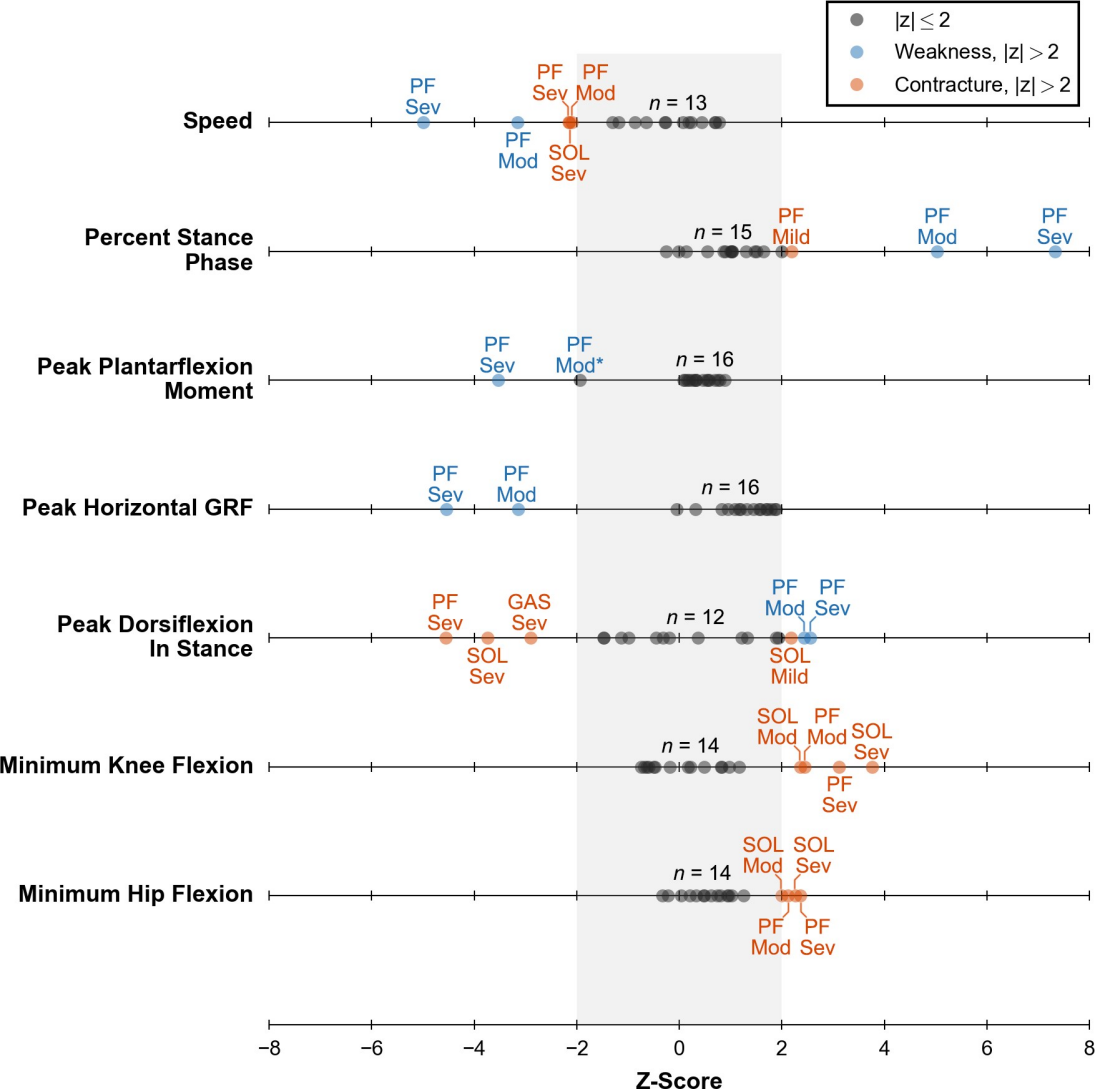
Z-scores of key kinematic and kinetic measures of gait for all deficit cases. Z-scores were computed using previous experimental data of individuals walking at a self-selected speed from Schwartz et al. [58]. All 18 cases (dots) are plotted for each measure. A black dot indicates any case within 2 SDs of normal (gray band). A blue or orange dot indicates either a weakness or contracture case, respectively, which is outside of 2 SDs. All of these cases are labeled with the affected muscle or muscles: soleus (SOL), gastrocnemius (GAS), or both (PF); and severity level: mild (Mild), moderate (Mod), or severe (Sev). One other case is labeled (see Peak Plantarflexion Moment, PF Mod*) as it was a clear outlier compared to other deficit cases. The count above each line (e.g., *n = 13*) indicates the number of unlabeled dots.

The optimization framework generated a stable gait in all deficit cases, highlighting its ability to change neural control in response to weakness and contracture (S2 Video). Furthermore, the model’s gait was robust to most deficits, and many cases had kinematic and kinetic measures within 2 SDs of walking at a self-selected speed (Fig 8, S3 Fig). By count, the most affected cases were moderate PF weakness, severe PF weakness, severe SOL contracture, and severe PF contracture. In the following sections, we detail the weakness and contracture cases that caused the model to adopt a gait with kinematic or kinetic features outside of 2 SDs of normal walking at a self-selected speed. We provide complete kinematic and kinetic trajectories for all simulations of muscle weakness and contracture in S4 Fig.

### Walking with plantarflexor weakness

**Fig 9:**
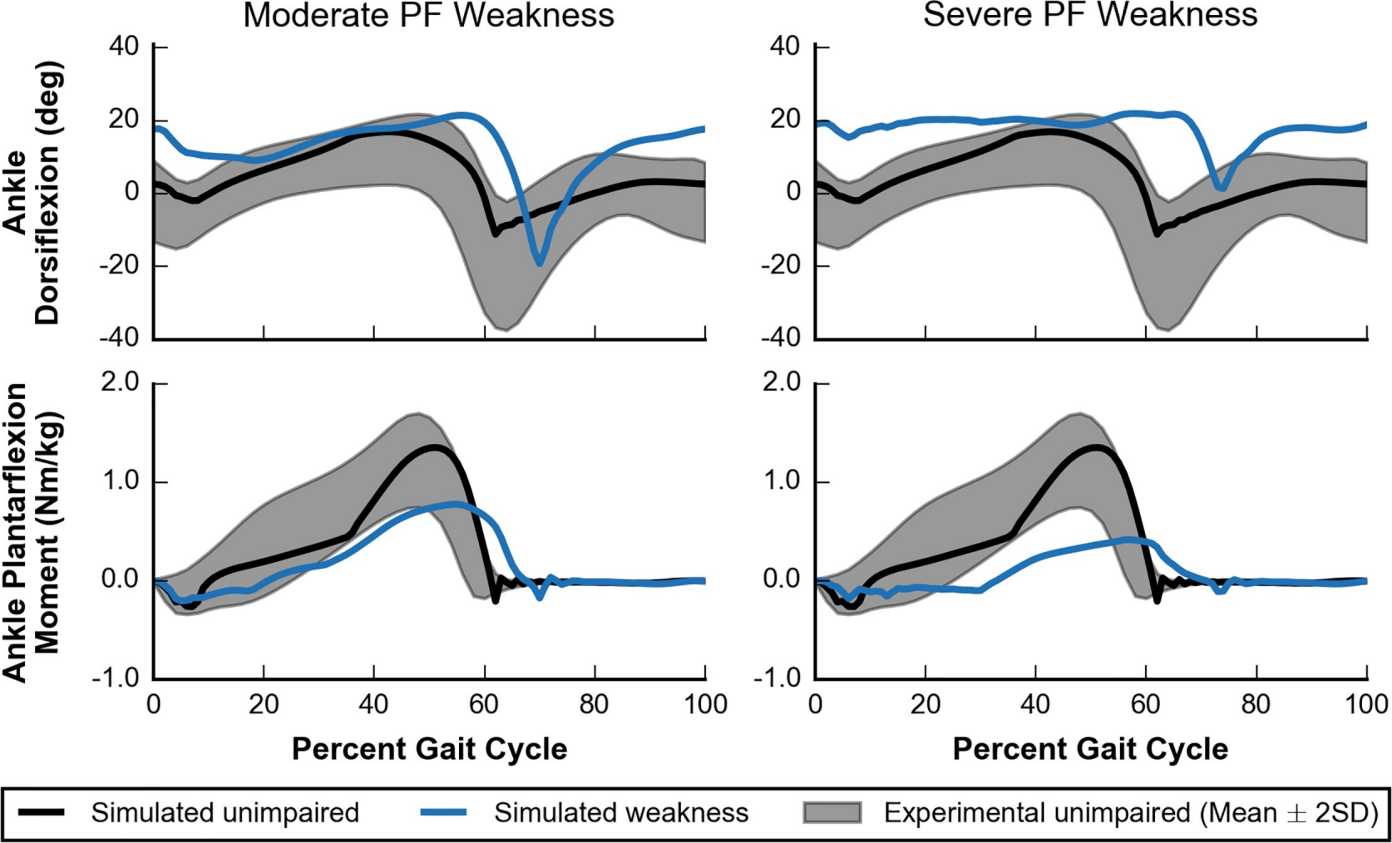
Ankle kinematics and kinetics for moderate and severe PF weakness. Simulated ankle kinematics and kinetics for unimpaired walking (black lines) and walking with PF weakness (blue lines) are compared to experimental data of unimpaired individuals (gray area) [58]. Ankle dorsiflexion (top) and ankle plantarflexion moment (bottom) are plotted for cases of moderate (left) and severe (right) PF weakness.

Plantarflexor weakness decreases the capacity of muscles to generate ankle plantarflexion moments, which are critical for generating forward propulsion during walking [1,2,17]. Our simulations supported this notion as plantarflexion moments during walking were most affected by moderate and severe PF weakness and minimally affected by SOL or GAS weakness only (Fig 8, third row). This suggests that in cases of weakness to only the SOL or GAS, the unimpaired plantarflexor had sufficient capacity to compensate for weakness in the other muscle. For moderate and severe PF weakness, peak ankle plantarflexion moment decreased by 43% and 69%, respectively, when compared to simulated unimpaired walking (Fig 9, bottom row). This highlights the reduced moment-generating capacity in these cases.

Other gait parameters, such as peak horizontal GRF, self-selected walking speed, and percent stance were abnormal for moderate and severe PF weakness as well (Fig 8). Compared to simulated unimpaired walking, peak horizontal GRF decreased by 55% and 70% for moderate and severe PF, respectively, which led to slower walking speeds of 1.01 m/s and 0.87 m/s, respectively. These decreases in gait speed in response to weakness are unsurprising given the direct relationship between the push-off phase and walking speed. Reduced walking speed is commonly observed in individuals with cerebral palsy [65], stroke [66], muscular dystrophy [67], Charcot-Marie-Tooth disease [68], and sarcopenia [69], where plantarflexor weakness is common. After accounting for these reduced walking speeds, the model spent an increased percentage of the gait cycle in stance phase, 69% and 74% with moderate and severe PF weakness, respectively. For comparison, simulated unimpaired walking at a slower speed of 0.75 m/s had a percent stance phase of only 63% (Fig 5). These increases in percent stance phase are consistent with trends seen in the pathological population. For example, patients with Charcot-Marie-Tooth disease who had a “plantar flexor strength deficit” were found to adopt a slower gait with an increased time spent in stance phase as compared to the gait chosen by patients without a strength deficit [70].

Dramatic kinematic compensations at the ankle were observed in cases of moderate and severe PF weakness. These models adopted a calcaneal, or “heel-walking”, gait, landing with substantially increased ankle dorsiflexion that was maintained throughout stance (Fig 9, top row). When compared with simulated unimpaired walking, the model with moderate or severe PF weakness had an increased ankle dorsiflexion at initial contact of 15° and 16°, respectively, and an increased average ankle dorsiflexion during stance of 7° and 11°, respectively. This type of gait is often observed in patients who have received either an intramuscular aponeurotic recession of the plantarflexors or Achilles tendon lengthening to correct contracture [71–73], and it is commonly thought that overlengthening during these procedures can lead to plantarflexor weakness [74,75]. Our simulations support the notion that plantarflexor weakness alone could cause calcaneal gait.

While it has been previously hypothesized that plantarflexor weakness may contribute to individuals adopting a crouch gait [19,72,73,75], our simulations did not support this hypothesis. Crouch gait is characterized by increased knee flexion during stance and commonly occurs with increased hip flexion during stance, but our optimization framework found gaits that did not require substantial plantarflexion moment and did not have increased knee and hip flexion during stance (Fig 8, bottom two rows). Our results also differ from previous work that observed a mild crouch with weakened plantarflexors [22], but a key difference was that our simulations did not have a constrained speed whereas the previous work had a prescribed speed of 1.25 m/s.

### Walking with plantarflexor contracture

**Fig 10:**
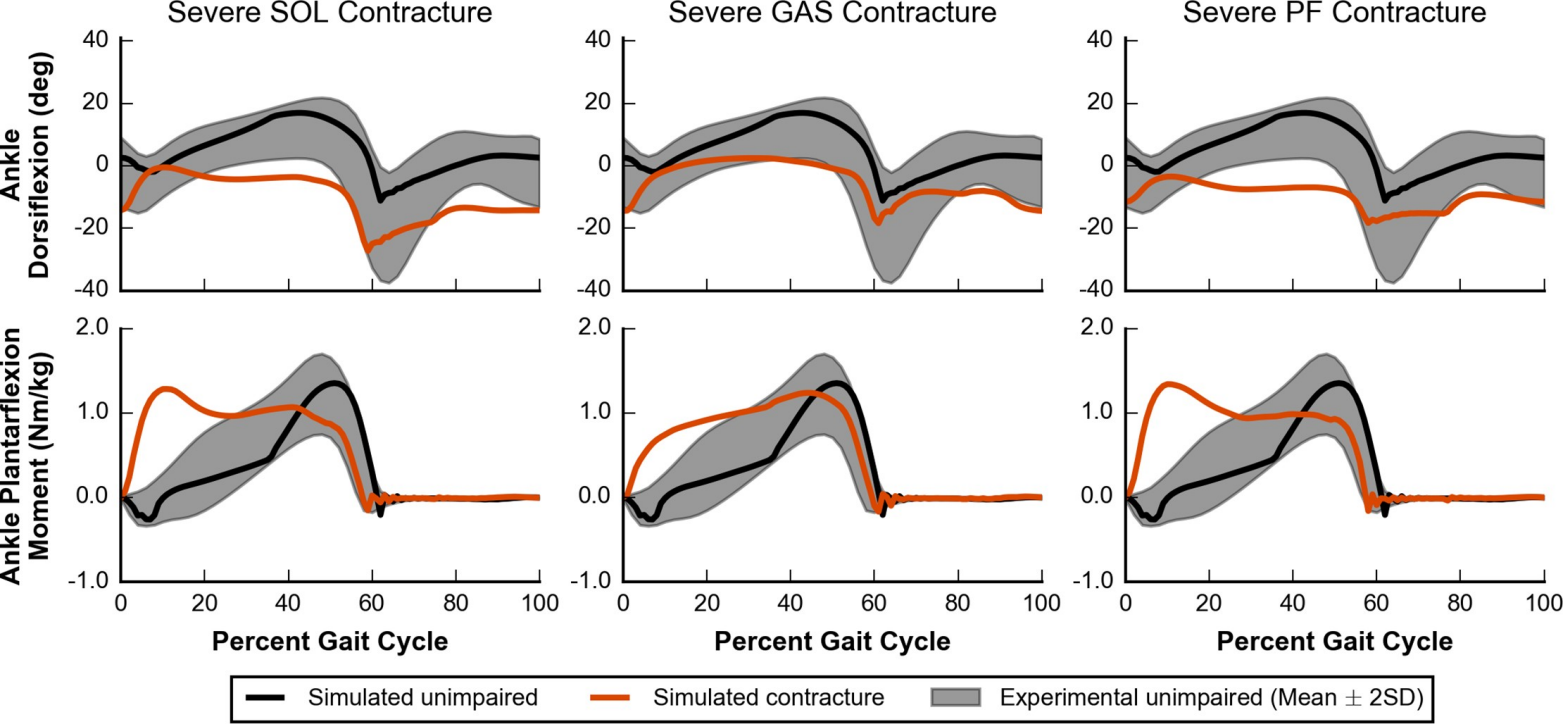
Ankle kinematics and kinetics for severe contracture. Simulated ankle kinematics and kinetics for unimpaired walking (black lines) and walking with severe contracture (orange lines) are compared to experimental data of unimpaired individuals (gray area) [58]. Ankle dorsiflexion (top) and ankle plantarflexion moment (bottom) are plotted for cases of SOL (left), GAS (middle), and PF (right) contracture.

Plantarflexor contracture increases passive ankle plantarflexion moments, shifting the ankle’s resting position to a more plantarflexed state and limiting ankle range of motion [11,13]. All cases of severe contracture (i.e., SOL, GAS, and PF) had significantly decreased peak values of dorsiflexion in stance (Fig 8, fifth row). SOL contracture caused a larger change (i.e., more plantarflexion in stance) than GAS contracture, likely due to the higher passive forces at the same percent contracture because the maximum isometric force of SOL is almost 70% larger than that of GAS (S1 Table). One case, mild SOL contracture, appeared to have an opposite effect, but we believe this is an anomaly as the overall ankle position trajectory did not differ much from the unimpaired case (S4 Fig, subfigure D). Models with severe contracture landed on their toes as the ankle was plantarflexed beyond 0° at initial contact and stayed on their toes throughout stance (Fig 10, top row). Equinus, or “toe-walking,” gait is commonly observed in individuals with cerebral palsy [76,77]. Plantarflexor contracture is thought to be a major contributing factor [11,78], and our results support this theory.

All cases of severe contracture had significantly increased plantarflexion moments during early stance (Fig 10, bottom row), which was expected since an equinus gait necessitates large plantarflexion moments at initial contact to prevent excessive dorsiflexion. In particular, severe SOL or PF contracture had a peak plantarflexion moment during early stance rather than during the push-off phase. Although peak values were largely unaffected, plantarflexion moments during the push-off phase decreased which led to a subsequent decrease in peak horizontal GRF; all contracture cases had a peak horizontal GRF that was between 2% and 23% lower than our simulated unimpaired case (S3 Fig, fourth row).

As severity of contracture increased, models adapted by walking slower and with a shorter stance phase. For the three slowest conditions, which included severe SOL, moderate PF, and severe PF contracture, the models walked at a significantly slower speed of 1.08 m/s with a slightly smaller percent stance phase of 59% (Fig 8, top two rows). This small decrease in percent stance phase agrees well with experimental data of individuals walking in equinus [63].

**Fig 11:**
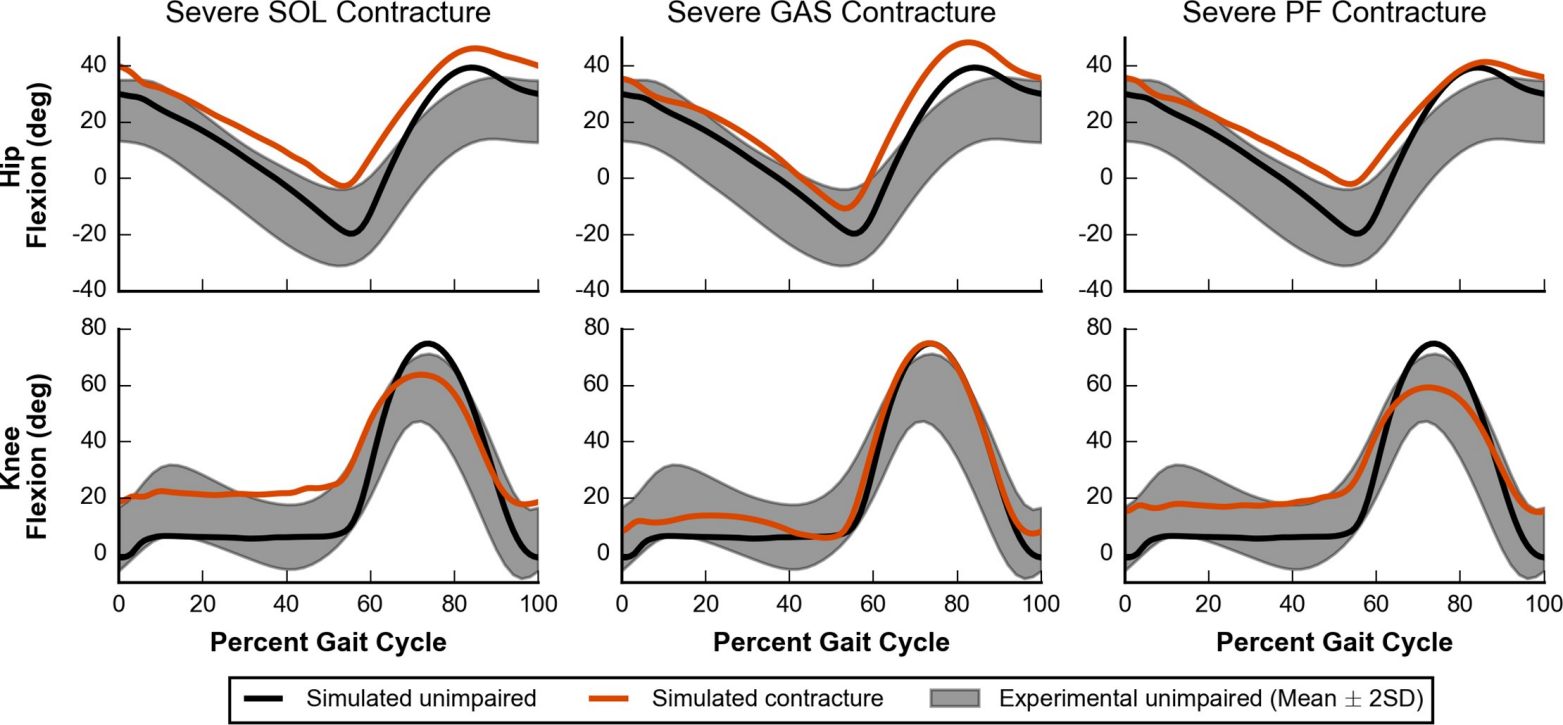
Hip and knee kinematics for severe contracture. Simulated hip and knee kinematics for unimpaired walking (black lines) and walking with severe contracture (orange lines) are compared to experimental data collected from unimpaired individuals (gray area) [58]. Hip flexion (top) and knee flexion (bottom) are plotted for cases of SOL (left), GAS (middle), and PF (right) contracture.

In cases of severe SOL, GAS, and PF contracture, the model adopted gaits that were more crouched throughout the whole stance phase, as measured by increased knee and hip flexion, when compared to simulated unimpaired walking (Fig 11). Cases of SOL and PF contracture were more crouched than cases of GAS contracture (S3 Fig, bottom two rows), highlighting that contracture in the plantarflexor muscles can lead to dramatic compensations at joints that they do not span.

## Discussion

Our model and simulation framework can robustly generate realistic simulations of gait at a range of speeds without tracking experimental data, as well as generate stable gaits in the presence of isolated plantarflexor deficits. By systematically introducing plantarflexor weakness or contracture, we were able to test hypotheses that are difficult or impossible to test in experiments due to co-occuring deficits. Our results showed that the model could walk without significant adaptations unless both plantarflexors were moderately or severely weakened. As expected, moderate and severe PF weakness reduced plantarflexion moment capacity, leading to a slower gait with increased percent time spent in stance phase. Our simulations also adopted a calcaneal gait, which supports previous hypotheses that PF weakness may contribute to this gait in patients [74,75]. Our results, however, did not support previous hypotheses that plantarflexor weakness contributes to individuals adopting a crouch gait. Although our work does not support the notion that plantarflexor weakness alone will lead to crouch gait, it does not rule out that it could be a contributing factor. For example, since plantarflexor weakness is often associated with weakness and contracture in other muscle groups, perhaps a combination of these deficits along with impaired neural control may lead to crouch gait.

Simulations of models with severe contracture of either the SOL or GAS, or of both PF showed significant adaptations at all lower joints. As expected, these cases caused the model to adopt an equinus gait due to increased passive forces in the plantarflexor muscles. We were also able to test if plantarflexor contracture alone could cause the model to adopt a crouch gait and elucidate possible relationships between plantarflexor contracture and crouch gait that would be difficult to isolate in experiments. Our results showed that SOL and PF contracture caused a more pronounced crouch gait as well as more severe equinus gait than GAS contracture. This leads to two conclusions. First, contracture in the plantarflexor muscles can lead to dramatic compensations at joints that they do not span. Second, because contracture of only GAS had the smallest effect, our results suggest that the crouched postures adopted by the model were affected less by the GAS directly flexing the knee and more by walking in equinus.

Our simulations of unimpaired gait captured many salient features of kinematics, kinetics, metabolics, and muscle activity. Over a range of prescribed speeds, our simulations captured key kinematic, kinetic, and metabolic trends observed in experiments. Without a target speed, our method found a self-selected gait that had similar kinematics, kinetics, and muscle activity to experimental data. However, this simulation did not accurately predict knee flexion during stance as the muscles crossing the knee generated a flexion moment rather than an extension moment. Our choice in objective function terms likely affected these results. First, we chose to minimize metabolic cost rather than a measure of fatigue in our objective function, and previous work has shown that minimizing metabolic cost led to simulations that avoided knee flexion during stance [25]. Other simulation work has shown that including a term to minimize knee joint load in the objective function may yield more realistic results [79]. Given this limitation, we focused our analysis of adaptations to plantarflexor weakness and contracture to measures that our method better predicted and that were not sensitive to events in early stance, such as those in Fig 8.

Our work builds upon previous work that developed methods to generate simulations of gait without tracking experimental data. We used a reflex-based controller based on work that showed these controllers could generate realistic gait patterns [21]. These controllers reduce the parameter space and provide stability through reflexes so that controller parameters could be optimized using single shooting methods [22–24]. Reflex-based controllers also add realistic constraints on the control space by modeling physiological feedback loops and sensors, such as muscle spindles that sense muscle length and velocity, Golgi tendon organs that sense tendon force [80], and neural delays [49,50]. However, these controllers cannot generate the full complexity of possible neural signals in humans, and this may have affected the solutions of our simulations of gait. Other trajectory optimization methods, such as direct collocation, solve for the open loop controls rather than using reflexes. This allows these methods to represent all possible complexities of neural signals [25], some of which may be non-physiological due to the lack of constraints on the control space.

## Limitations and future work

Simplifications in the model may have affected the results of our simulations. The model was constrained to sagittal plane motion and consequently lacked degrees of freedom, such as hip abduction, pelvis list, and pelvis rotation, which allow for out-of-plane motions. This made foot clearance more difficult and was likely the cause of compensations in the sagittal plane that increased knee and hip flexion during swing. For the models with deficits, these planar constraints did not allow for commonly observed compensations that are out of the sagittal plane, such as hip circumduction [81,82]. Furthermore, our model lacked degrees of freedom above the pelvis, and future studies could add these muscles and investigate compensations at the trunk level. The Hill-type musculotendon actuators also have limitations, such as overestimating length changes and velocities because of their simplified geometry [43,44]. The metabolic model implementation used in this study had been validated for use in studying running [52] and now has been validated in for walking in this study, and future work to validate this metabolic model for other uses will be important to gain more confidence in this model.

Although we chose to model weakness by decreasing $F_{o}^{m}$ and contracture by decreasing $l_{o}^{m}$, there are other ways to model these deficits. For instance, weakness could also be modeled as decreased maximum muscle excitation, which may be important in cases such as stroke [83], and contracture could also be modeled as decreased tendon slack length [84] or increased passive muscle stiffness [85]. These issues may interact with other co-occurring problems in the sensory and neural systems, such as in Charcot-Marie-Tooth disease [86], and introducing models of these deficits would allow future research to study this interaction. These additional modeling approaches may lead to different gait compensations, and future work to test sensitivity of our results to variations in modeling choices would be valuable.

While our objective function contained terms that are thought to capture high-level goals of walking, such as minimizing cost of transport [51] while maintaining head stability [55] (see Methods), it may not comprehensively represent goals of human walking, especially amongst individuals with gait pathologies. For example, some individuals may choose a trade-off between metabolic efficiency and joint loading or stability to perturbations. Furthermore, the relative trade-off between these competing objectives likely varies between individuals.

Due to the non-convex nature of the optimization problem and the stochastic nature of our optimizer, the solutions provided here are unlikely to be global minima. To gain confidence in our solutions, we followed previous work that used multiple optimizations from the same seed and restarted optimizations from the previous best result [24,57], and furthermore, we demonstrated how our solution was robust to different initial seeds (see section on “Validating the model’s gait at self-selected speed”). This strategy, however, was computationally expensive, and further work is needed to better search this complex, high-dimensional space. Since a good starting guess can be important for finding reasonable results, we provide our results to assist other researchers in future studies. Using direct collocation in future studies to generate these types of simulations could also be useful to overcome the high computational cost of single shooting methods [25].

Although this work presents a framework for studying how isolated changes in muscle parameters may lead to changes in gait, future work on how gait adapts with changes in neural control will further our understanding of pathologic gait. Reflex-based controllers, like the one used here, are useful to ensure that the optimization problem was tractable, but a controller like this cannot predict changes in the underlying control structure. Recent advances in the field of reinforcement learning may be able to generate robust simulations with control patterns that do not have a specified and simplified control structure [87,88].

## Conclusion

We present a model and optimization framework suitable for studying sagittal plane adaptations during walking due to muscular deficits. We first validated that, over a wide range of speeds, our model and framework captured many salient features of gait. This framework was then used to study how the model would adapt its gait when plantarflexor weakness or contracture was introduced. Severe plantarflexor weakness caused the model to adopt a calcaneal gait without crouching, while severe plantarflexor contracture caused the model to adopt an equinus gait. Our simulations also showed that contracture of only SOL or both PF caused the model to walk in a more crouched posture than if contracture was applied to only GAS, which suggests that walking in equinus is a major contributor to the observed crouch gait rather than force from the GAS directly flexing the knee. We provide our results freely at https://simtk.org/projects/pfdeficitsgait and software at https://scone.software so that others can easily extend this work.

## Supporting information

S1 Fig Kinematic and kinetic trajectories of simulated data and experimental data at prescribed speeds between 0.50 m/s and 2.00 m/s. (PDF)

S2 Fig Kinematic and kinetic trajectories for all three self-selected gait seeds (black lines) against experimental data (gray area). (PDF)

S3 Fig Key kinematic and kinetic parameters for all deficits. This plot accompanies Fig 8 and shows key parameters for walking with simulated weakness (blue) and contracture (orange) of the SOL, GAS, or both (PF) along with simulated healthy gait (dotted line). Increasing color intensity indicates increasing deficit severity.
(PDF)

S4 Fig Kinematic and kinetic trajectories of all simulated deficits. (PDF)

S1 Table Muscle parameters for the unimpaired musculoskeletal model. (PDF)

S2 Table Initial Covariance Matrix Adaptation Evolutionary Strategy (CMA-ES) standard deviation for each free parameter. (PDF)

S3 Table Muscle activity comparisons between simulations and *Perry and Burnfield*, *2010* for Fig 7. (PDF)

S1 Video Highlights. (MP4)

S2 Video Compilation of all simulations. (MP4)

## References

[1] Neptune RR, Kautz SA, Zajac FE. Contributions of the individual ankle plantar flexors to support, forward progression and swing initiation during walking. J Biomech. 2001;34: 1387–1398. 10.1016/s0021-9290(01)00105-1 11672713

[2] Liu MQ, Anderson FC, Schwartz MH, Delp SL. Muscle contributions to support and progression over a range of walking speeds. J Biomech. 2008;41: 3243–3252. 10.1016/j.jbiomech.2008.07.031 18822415 PMC4423744

[3] Handsfield GG, Meyer CH, Abel MF, Blemker SS. Heterogeneity of muscle sizes in the lower limbs of children with cerebral palsy. Muscle Nerve. 2016;53: 933–945. 10.1002/mus.24972 26565390

[4] Noble JJ, Fry NR, Lewis AP, Keevil SF, Gough M, Shortland AP. Lower limb muscle volumes in bilateral spastic cerebral palsy. Brain Dev. 2014;36: 294–300. 10.1016/j.braindev.2013.05.008 23790825

[5] Barber L, Hastings-Ison T, Baker R, Barrett R, Lichtwark G. Medial gastrocnemius muscle volume and fascicle length in children aged 2 to 5 years with cerebral palsy. Dev Med Child Neurol. 2011;53: 543–548. 10.1111/j.1469-8749.2011.03913.x 21506995

[6] Kim CM, Eng JJ. The Relationship of Lower-Extremity Muscle Torque to Locomotor Performance in People With Stroke. Phys Ther. 2003;83: 49–57. 10.1093/ptj/83.1.49 12495412

[7] Hoogerwaard EM, Bakker E, Ippel PF, Oosterwijk JC, Majoor-Krakauer DF, Leschot NJ, et al Signs and symptoms of Duchenne muscular dystrophy and Becker muscular dystrophy among carriers in The Netherlands: a cohort study. Lancet. 1999;353: 2116–2119. 10.1016/s0140-6736(98)10028-4 10382696

[8] Bushby K, Finkel R, Birnkrant DJ, Case LE, Clemens PR, Cripe L, et al Diagnosis and management of Duchenne muscular dystrophy, part 1: diagnosis, and pharmacological and psychosocial management. Lancet Neurol. 2010;9: 77–93. 10.1016/S1474-4422(09)70271-6 19945913

[9] Sabir M, Lyttle D. Pathogenesis of pes cavus in Charcot-Marie-Tooth disease. Clin Orthop Relat Res. 1983; 173–178. 6839584

[10] Barber L, Barrett R, Lichtwark G. Passive muscle mechanical properties of the medial gastrocnemius in young adults with spastic cerebral palsy. J Biomech. 2011;44: 2496–2500. 10.1016/j.jbiomech.2011.06.008 21762920

[11] Mathewson MA, Ward SR, Chambers HG, Lieber RL. High resolution muscle measurements provide insights into equinus contractures in patients with cerebral palsy. J Orthop Res. 2015;33: 33–39. 10.1002/jor.22728 25242618 PMC4343320

[12] Lin P-Y, Yang Y-R, Cheng S-J, Wang R-Y. The Relation Between Ankle Impairments and Gait Velocity and Symmetry in People With Stroke. Arch Phys Med Rehabil. 2006;87: 562–568. 10.1016/j.apmr.2005.12.042 16571398

[13] Gao F, Grant TH, Roth EJ, Zhang L-Q. Changes in Passive Mechanical Properties of the Gastrocnemius Muscle at the Muscle Fascicle and Joint Levels in Stroke Survivors. Arch Phys Med Rehabil. 2009;90: 819–826. 10.1016/j.apmr.2008.11.004 19406302

[14] Mercuri E, Bushby K, Ricci E, Birchall D, Pane M, Kinali M, et al Muscle MRI findings in patients with limb girdle muscular dystrophy with calpain 3 deficiency (LGMD2A) and early contractures. Neuromuscul Disord. 2005;15: 164–171. 10.1016/j.nmd.2004.10.008 15694138

[15] Bushby K, Finkel R, Birnkrant DJ, Case LE, Clemens PR, Cripe L, et al Diagnosis and management of Duchenne muscular dystrophy, part 2: implementation of multidisciplinary care. Lancet Neurol. 2010;9: 177–189. 10.1016/S1474-4422(09)70272-8 19945914

[16] Berger MJ, Doherty TJ. Sarcopenia: Prevalence, Mechanisms, and Functional Consequences. Interdiscipl Top Gerontol. 2010;37: 94–114. 10.1159/000319997 20703058

[17] Sutherland DH, Cooper L, Daniel D. The role of the ankle plantar flexors in normal walking. JBJS. 1980;62: 354–363. 7364808

[18] Steele KM, Seth A, Hicks JL, Schwartz MS, Delp SL. Muscle contributions to support and progression during single-limb stance in crouch gait. J Biomech. 2010;43: 2099–2105. 10.1016/j.jbiomech.2010.04.003 20493489 PMC2914221

[19] Steele KM, van der Krogt MM, Schwartz MH, Delp SL. How much muscle strength is required to walk in a crouch gait? J Biomech. 2012;45: 2564–2569. 10.1016/j.jbiomech.2012.07.028 22959837 PMC3524281

[20] van der Krogt MM, Bar-On L, Kindt T, Desloovere K, Harlaar J. Neuro-musculoskeletal simulation of instrumented contracture and spasticity assessment in children with cerebral palsy. J Neuroeng Rehabil. 2016;13: 64 10.1186/s12984-016-0170-5 27423898 PMC4947289

[21] Geyer H, Herr H. A Muscle-Reflex Model That Encodes Principles of Legged Mechanics Produces Human Walking Dynamics and Muscle Activities. IEEE Trans Neural Syst Rehabil Eng. 2010;18: 263–273. 10.1109/TNSRE.2010.2047592 20378480

[22] Wang JM, Hamner SR, Delp SL, Koltun V. Optimizing Locomotion Controllers Using Biologically-Based Actuators and Objectives. ACM Trans Graph. 2012;31: 25 10.1145/2185520.2185521 26251560 PMC4523558

[23] Dorn TW, Wang JM, Hicks JL, Delp SL. Predictive Simulation Generates Human Adaptations during Loaded and Inclined Walking. PLoS One. 2015;10: e0121407 10.1371/journal.pone.0121407 25830913 PMC4382289

[24] Song S, Geyer H. A neural circuitry that emphasizes spinal feedback generates diverse behaviours of human locomotion. J Physiol. 2015;593: 3493–3511. 10.1113/JP270228 25920414 PMC4560581

[25] Ackermann M, van den Bogert AJ. Optimality principles for model-based prediction of human gait. J Biomech. Elsevier; 2010;43: 1055–1060. 10.1016/j.jbiomech.2009.12.012 20074736 PMC2849893

[26] Miller RH, Umberger BR, Hamill J, Caldwell GE. Evaluation of the minimum energy hypothesis and other potential optimality criteria for human running. Proc R Soc B Biol Sci. 2012;279: 1498–1505. 10.1098/rspb.2011.2015 22072601 PMC3282349

[27] van den Bogert AJ, Blana D, Heinrich D. Implicit methods for efficient musculoskeletal simulation and optimal control. Procedia IUTAM. 2011;2: 297–316. 10.1016/j.piutam.2011.04.027 22102983 PMC3217276

[28] Handford ML, Srinivasan M. Robotic lower limb prosthesis design through simultaneous computer optimizations of human and prosthesis costs. Sci Rep. 2016;6: 19983 10.1038/srep19983 26857747 PMC4746571

[29] Song S, Geyer H. Predictive neuromechanical simulations indicate why walking performance declines with ageing. J Physiol. 2018;596: 1199–1210. 10.1113/JP275166 29344967 PMC5878225

[30] Geijtenbeek T. SCONE: Open Source Software for Predictive Simulation of Biological Motion. J Open Source Softw. 2019;4: 1421 10.21105/joss.01421

[31] Delp SL, Anderson FC, Arnold AS, Loan P, Habib A, John CT, et al OpenSim: open-source software to create and analyze dynamic simulations of movement. IEEE Trans Biomed Eng. 2007;54: 1940–1950. 10.1109/TBME.2007.901024 18018689

[32] Seth A, Hicks JL, Uchida TK, Habib A, Dembia CL, Dunne JJ, et al OpenSim: Simulating musculoskeletal dynamics and neuromuscular control to study human and animal movement. PLOS Comput Biol. 2018;14: 1–20. 10.1371/journal.pcbi.1006223 30048444 PMC6061994

[33] Delp SL, Loan JP, Hoy MG, Zajac FE, Topp EL, Rosen JM. An interactive graphics-based model of the lower extremity to study orthopaedic surgical procedures. IEEE Trans Biomed Eng. 1990;37: 757–767. 10.1109/10.102791 2210784

[34] Thelen DG. Adjustment of Muscle Mechanics Model Parameters to Simulate Dynamic Contractions in Older Adults. J Biomech Eng. 2003;125: 70–77. 10.1115/1.1531112 12661198

[35] Rajagopal A, Dembia CL, DeMers MS, Delp DD, Hicks JL, Delp SL. Full-Body Musculoskeletal Model for Muscle-Driven Simulation of Human Gait. IEEE Trans Biomed Eng. 2016;63: 2068–2079. 10.1109/TBME.2016.2586891 27392337 PMC5507211

[36] Handsfield GG, Meyer CH, Hart JM, Abel MF, Blemker SS. Relationships of 35 lower limb muscles to height and body mass quantified using MRI. J Biomech. 2014;47: 631–638. 10.1016/j.jbiomech.2013.12.002 24368144

[37] Millard M, Uchida T, Seth A, Delp SL. Flexing Computational Muscle: Modeling and Simulation of Musculotendon Dynamics. J Biomech Eng. 2013;135: 21005–21011. 10.1115/1.4023390 23445050 PMC3705831

[38] Arnold EM, Hamner SR, Seth A, Millard M, Delp SL. How muscle fiber lengths and velocities affect muscle force generation as humans walk and run at different speeds. J Exp Biol. 2013; 10.1242/jeb.075697 23470656 PMC3656509

[39] Whittington B, Silder A, Heiderscheit B, Thelen DG. The contribution of passive-elastic mechanisms to lower extremity joint kinetics during human walking. Gait Posture. 2008;27: 628–634. 10.1016/j.gaitpost.2007.08.005 17928228 PMC2505349

[40] Ward SR, Eng CM, Smallwood LH, Lieber RL. Are Current Measurements of Lower Extremity Muscle Architecture Accurate? Clin Orthop Relat Res. 2009;467: 1074–1082. 10.1007/s11999-008-0594-8 18972175 PMC2650051

[41] Arnold EM, Ward SR, Lieber RL, Delp SL. A Model of the Lower Limb for Analysis of Human Movement. Ann Biomed Eng. 2010;38: 269–279. 10.1007/s10439-009-9852-5 19957039 PMC2903973

[42] Thelen DG, Chumanov ES, Best TM, Swanson SC, Heiderscheit BC. Simulation of biceps femoris musculotendon mechanics during the swing phase of sprinting. Med Sci Sports Exerc. 2005;37: 1931–1938. 10.1249/01.mss.0000176674.42929.de 16286864

[43] Blemker SS, Delp SL. Rectus femoris and vastus intermedius fiber excursions predicted by three-dimensional muscle models. J Biomech. 2006;39: 1383–1391. 10.1016/j.jbiomech.2005.04.012 15972213

[44] Chen X, Sanchez GN, Schnitzer MJ, Delp SL. Changes in sarcomere lengths of the human vastus lateralis muscle with knee flexion measured using in vivo microendoscopy. J Biomech. 2016;49: 2989–2994. 10.1016/j.jbiomech.2016.07.013 27481293 PMC5507365

[45] Hunt KH, Crossley FRE. Coefficient of Restitution Interpreted as Damping in Vibroimpact. J Appl Mech. 1975;42: 440–445. 10.1115/1.3423596

[46] Sherman MA, Seth A, Delp SL. Simbody: multibody dynamics for biomedical research. Procedia IUTAM. 2011;2: 241–261. 10.1016/J.PIUTAM.2011.04.023 25866705 PMC4390141

[47] Ziv I, Blackburn N, Rang M, Koreska J. Muscle growth in normal and spastic mice. Dev Med Child Neurol. 1984;26: 94–99. 10.1111/j.1469-8749.1984.tb04412.x 6698331

[48] Delp SL, Statler K, Carroll NC. Preserving plantar flexion strength after surgical treatment for contracture of the triceps surae: A computer simulation study. J Orthop Res. 1995;13: 96–104. 10.1002/jor.1100130115 7853110

[49] Duysens J, Trippel M, Horstmann G, Dietz V. Gating and reversal of reflexes in ankle muscles during human walking. Exp Brain Res. 1990;82: 351–358. 10.1007/bf00231254 2286237

[50] Frijns CJM, Laman DM, van Duijn MAJ, van Duijn H. Normal values of patellar and ankle tendon reflex latencies. Clin Neurol Neurosurg. 1997;99: 31–36. 10.1016/s0303-8467(96)00593-8 9107465

[51] Selinger JC, O’Connor SM, Wong JD, Donelan JM. Humans Can Continuously Optimize Energetic Cost during Walking. Curr Biol. 2015;25: 2452–2456. 10.1016/j.cub.2015.08.016 26365256

[52] Uchida TK, Hicks JL, Dembia CL, Delp SL. Stretching Your Energetic Budget: How Tendon Compliance Affects the Metabolic Cost of Running. PLoS One. 2016;11: 1–19. 10.1371/journal.pone.0150378 26930416 PMC4773147

[53] Umberger BR, Gerritsen KGM, Martin PE. A model of human muscle energy expenditure. Comput Methods Biomech Biomed Engin. 2003;6: 99–111. 10.1080/1025584031000091678 12745424

[54] Bril B, Ledebt A. Head Coordination as a Means to Assist Sensory Integration in Learning to Walk. Neurosci Biobehav Rev. 1998;22: 555–563. 10.1016/S0149-7634(97)00044-4 9595569

[55] Menz HB, Lord SR, Fitzpatrick RC. Acceleration patterns of the head and pelvis when walking on level and irregular surfaces. Gait Posture. 2003;18: 35–46. 10.1016/S0966-6362(02)00159-5 12855299

[56] Hansen N, Kern S. Evaluating the CMA Evolution Strategy on Multimodal Test Functions. In: Yao X, Burke EK, Lozano JA, Smith J, Merelo-Guervós JJ, Bullinaria JA, et al, editors. Parallel Problem Solving from Nature—PPSN VIII Berlin, Heidelberg: Springer Berlin Heidelberg; 2004 pp. 282–291.

[57] Ong CF, Hicks JL, Delp SL. Simulation-Based Design for Wearable Robotic Systems: An Optimization Framework for Enhancing a Standing Long Jump. IEEE Trans Biomed Eng. 2016;63: 894–903. 10.1109/TBME.2015.2463077 26258930 PMC5507207

[58] Schwartz MH, Rozumalski A, Trost JP. The effect of walking speed on the gait of typically developing children. J Biomech. 2008;41: 1639–1650. 10.1016/j.jbiomech.2008.03.015 18466909

[59] Ralston HJ. Energy-speed relation and optimal speed during level walking. Int Zeitschrift für Angew Physiol einschließlich Arbeitsphysiologie. 1958;17: 277–283. 10.1007/BF00698754 13610523

[60] Martin PE, Rothstein DE, Larish DD. Effects of age and physical activity status on the speed-aerobic demand relationship of walking. J Appl Physiol. 1992;73: 200–206. 10.1152/jappl.1992.73.1.200 1506370

[61] Browning RC, Baker EA, Herron JA, Kram R. Effects of obesity and sex on the energetic cost and preferred speed of walking. J Appl Physiol. 2006;100: 390–398. 10.1152/japplphysiol.00767.2005 16210434

[62] Wren TAL, Do KP, Rethlefsen SA, Healy B. Cross-correlation as a method for comparing dynamic electromyography signals during gait. J Biomech. 2006;39: 2714–2718. 10.1016/j.jbiomech.2005.09.006 16219314

[63] Perry J, Burnfield JM. Gait Analysis: Normal and pathological function. SLACK Inc.; 2010.

[64] Nene A, Byrne C, Hermens H. Is rectus femoris really a part of quadriceps?: Assessment of rectus femoris function during gait in able-bodied adults. Gait Posture. 2004;20: 1–13. 10.1016/S0966-6362(03)00074-2 15196513

[65] Abel MF, Damiano DL. Strategies for increasing walking speed in diplegic cerebral palsy. J Pediatr Orthop. 1996;16: 753–758. 10.1097/00004694-199611000-00010 8906647

[66] Turnbull GI, Charteris J, Wall JC. A comparison of the range of walking speeds between normal and hemiplegic subjects. Scand J Rehabil Med. 1995;27: 175–182. 8602480

[67] Khodadadeh S, McClelland MR, Patrick JH. Variations of Gait Parameters in Duchenne Muscular Dystrophy. Proc Inst Mech Eng Part H J Eng Med. 1990;204: 241–243. 10.1243/PIME_PROC_1990_204_262_02 2090127

[68] Newman CJ, Walsh M, O’Sullivan R, Jenkinson A, Bennett D, Lynch B, et al The characteristics of gait in Charcot-Marie-Tooth disease types I and II. Gait Posture. 2007;26: 120–127. 10.1016/j.gaitpost.2006.08.006 17010610

[69] Beijersbergen CMI, Granacher U, Vandervoort AA, DeVita P, Hortobágyi T. The biomechanical mechanism of how strength and power training improves walking speed in old adults remains unknown. Ageing Res Rev. 2013;12: 618–627. 10.1016/j.arr.2013.03.001 23501431

[70] Don R, Serrao M, Vinci P, Ranavolo A, Cacchio A, Ioppolo F, et al Foot drop and plantar flexion failure determine different gait strategies in Charcot-Marie-Tooth patients. Clin Biomech. 2007;22: 905–916. 10.1016/J.CLINBIOMECH.2007.06.002 17686557

[71] Dreher T, Buccoliero T, Wolf SI, Heitzmann D, Gantz S, Braatz F, et al Long-term results after gastrocnemius-soleus intramuscular aponeurotic recession as a part of multilevel surgery in spastic diplegic cerebral palsy. J Bone Joint Surg Am. 2012;94: 627–637. 10.2106/JBJS.K.00096 22488619

[72] Dietz FR, Albright JC, Dolan L. Medium-term follow-up of Achilles tendon lengthening in the treatment of ankle equinus in cerebral palsy. Iowa Orthop J. 2006;26: 27–32. 16789444 PMC1888588

[73] Segal LS, Thomas SE, Mazur JM, Mauterer M. Calcaneal gait in spastic diplegia after heel cord lengthening: a study with gait analysis. J Pediatr Orthop. 1989;9: 697–701. 10.1097/01241398-198911000-00013 2600179

[74] Garbarino JL, Clancy M. A geometric method of calculating tendo Achillis lengthening. J Pediatr Orthop. 1985;5: 573–576. 10.1097/01241398-198509000-00013 4044816

[75] Gage JR. Surgical treatment of knee dysfunction in cerebral palsy. Clin Orthop Relat Res. 1990; 45–54. 2317990

[76] Fulford GE. Surgical management of ankle and foot deformities in cerebral palsy. Clin Orthop Relat Res. 1990; 55–61. 2317991

[77] Wren TAL, Rethlefsen S, Kay RM. Prevalence of specific gait abnormalities in children with cerebral palsy: influence of cerebral palsy subtype, age, and previous surgery. J Pediatr Orthop. 2005;25: 79–83. 10.1097/00004694-200501000-00018 15614065

[78] Wren TAL, Do KP, Kay RM. Gastrocnemius and soleus lengths in cerebral palsy equinus gait—differences between children with and without static contracture and effects of gastrocnemius recession. J Biomech. 2004;37: 1321–1327. 10.1016/j.jbiomech.2003.12.035 15275839

[79] DeMers MS, Pal S, Delp SL. Changes in tibiofemoral forces due to variations in muscle activity during walking. J Orthop Res. Wiley Online Library; 2014;32: 769–776. 10.1002/jor.22601 24615885 PMC4409006

[80] Kistemaker DA, Van Soest AJK, Wong JD, Kurtzer I, Gribble PL. Control of position and movement is simplified by combined muscle spindle and Golgi tendon organ feedback. J Neurophysiol. American Physiological Society Bethesda, MD; 2012;109: 1126–1139. 10.1152/jn.00751.2012 23100138 PMC3569141

[81] Gage JR, Novacheck TF. An update on the treatment of gait problems in cerebral palsy. J Pediatr Orthop B. 2001;10: 265–274. 10.1097/00009957-200110000-00001 11727367

[82] Kim CM, Eng JJ. Magnitude and pattern of 3D kinematic and kinetic gait profiles in persons with stroke: relationship to walking speed. Gait Posture. 2004;20: 140–146. 10.1016/j.gaitpost.2003.07.002 15336283 PMC3167865

[83] Newham DJ, Hsiao SF. Knee muscle isometric strength, voluntary activation and antagonist co-contraction in the first six months after stroke. Disabil Rehabil. 2001;23: 379–386. 10.1080/0963828001006656 11394588

[84] Herbert RD, Crosbie J. Rest length and compliance of non-immobilised and immobilised rabbit soleus muscle and tendon. Eur J Appl Physiol Occup Physiol. 1997;76: 472–479. 10.1007/s004210050277 9367288

[85] Smith LR, Lee KS, Ward SR, Chambers HG, Lieber RL. Hamstring contractures in children with spastic cerebral palsy result from a stiffer extracellular matrix and increased in vivo sarcomere length. J Physiol. 2011;589: 2625–2639. 10.1113/jphysiol.2010.203364 21486759 PMC3115830

[86] Pareyson D, Scaioli V, Laurà M. Clinical and electrophysiological aspects of Charcot-Marie-Tooth disease. NeuroMolecular Med. 2006;8: 3–22. 10.1385/NMM:8:1:123 16775364

[87] Heess N, TB D, Sriram S, Lemmon J, Merel J, Wayne G, et al Emergence of Locomotion Behaviours in Rich Environments. 2017; Available: arxiv:1707.02286

[88] Kidziński Ł, Mohanty SP, Ong CF, Hicks JL, Carroll SF, Levine S, et al Learning to Run Challenge: Synthesizing Physiologically Accurate Motion Using Deep Reinforcement Learning. In: Escalera S, Weimer M, editors. The NIPS ‘17 Competition: Building Intelligent Systems Cham: Springer International Publishing; 2018 pp. 101–120.

