# Supplementary material for: Predicting gait adaptations due to ankle plantarflexor muscle weakness and contracture using physics-based musculoskeletal simulations
Source: PLoS Comput Biol. 2019 Oct 7;15(10):e1006993. doi: 10.1371/journal.pcbi.1006993 (PMC6797212; doi:10.1371/journal.pcbi.1006993)
Supplement: S1 Fig — (PDF) [file pcbi.1006993.s001.pdf]

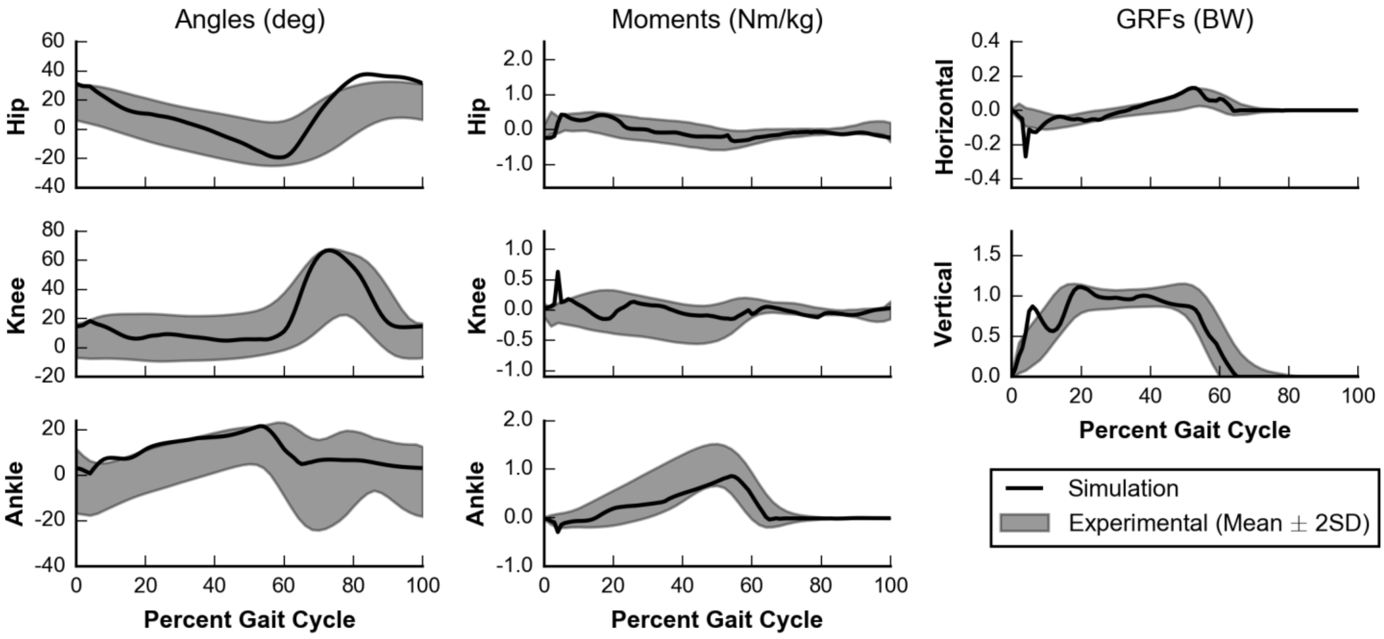

S1A Fig. Simulated walking at 0.55 m/s vs experimental data at 0.50 ± 0.20 m/s. To facilitate comparison, normalized speed from experimental data was converted using the model's leg length (0.856 m).

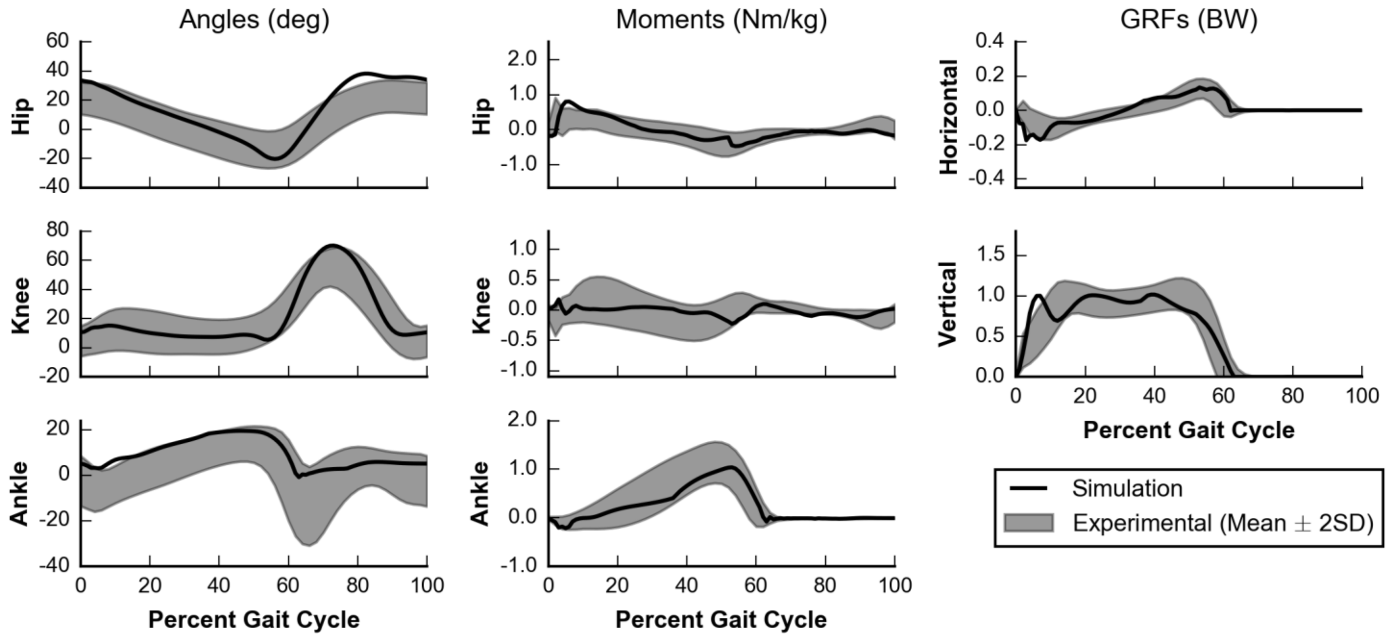

S1B Fig. Simulated walking at 0.79 m/s vs experimental data at 0.84 ± 0.16 m/s. To facilitate comparison, normalized speed from experimental data was converted using the model's leg length (0.856 m).

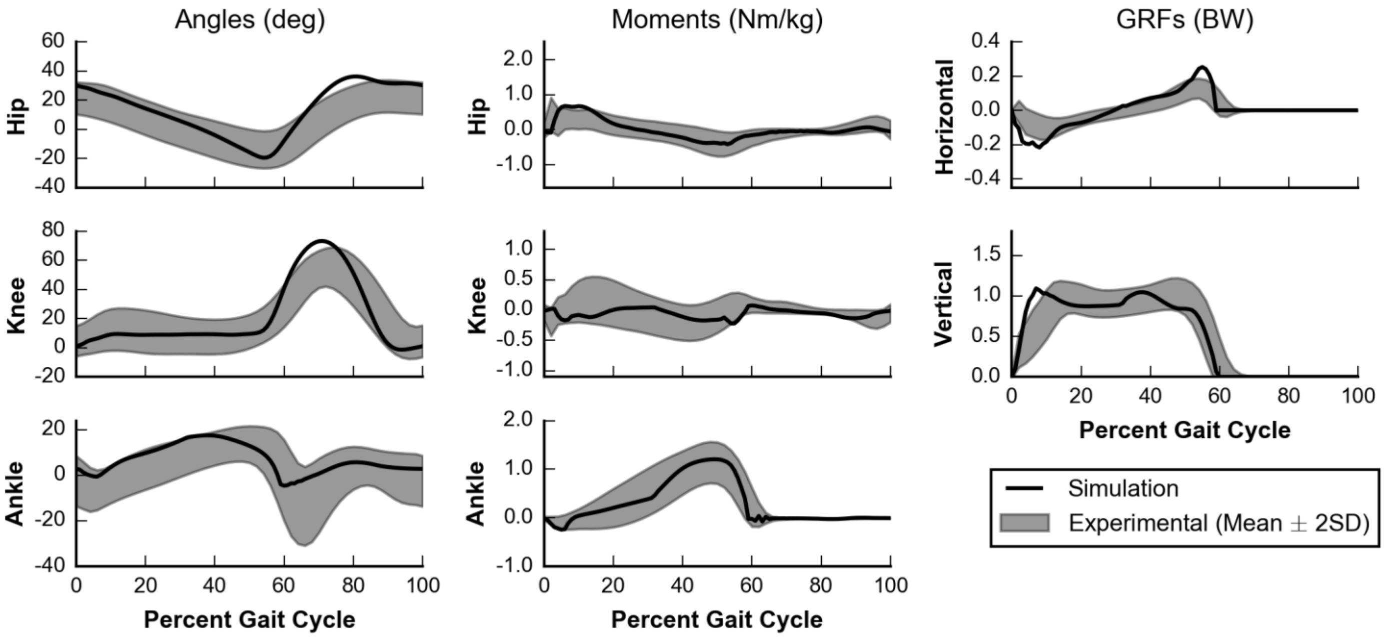

S1C Fig. Simulated walking at 1.04 m/s vs experimental data at 0.84 ± 0.16 m/s. To facilitate comparison, normalized speed from experimental data was converted using the model's leg length (0.856 m).

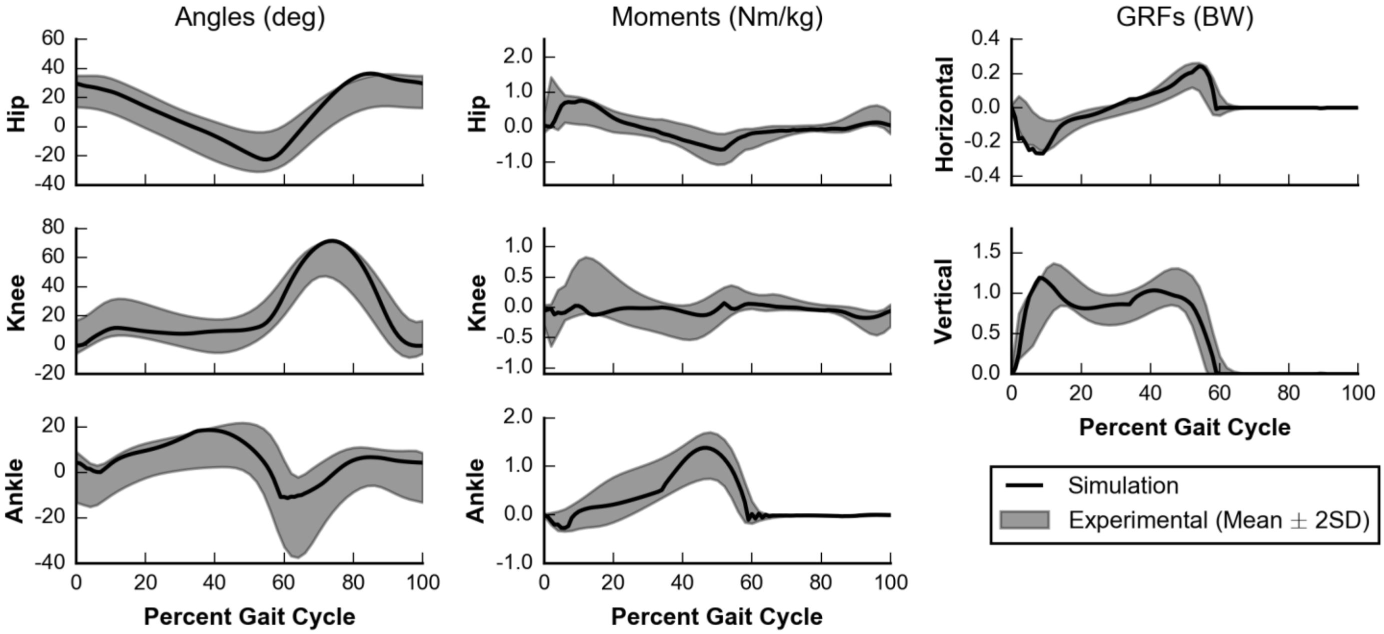

S1D Fig. Simulated walking at 1.22 m/s vs experimental data at 1.24 ± 0.15 m/s. To facilitate comparison, normalized speed from experimental data was converted using the model's leg length (0.856 m).

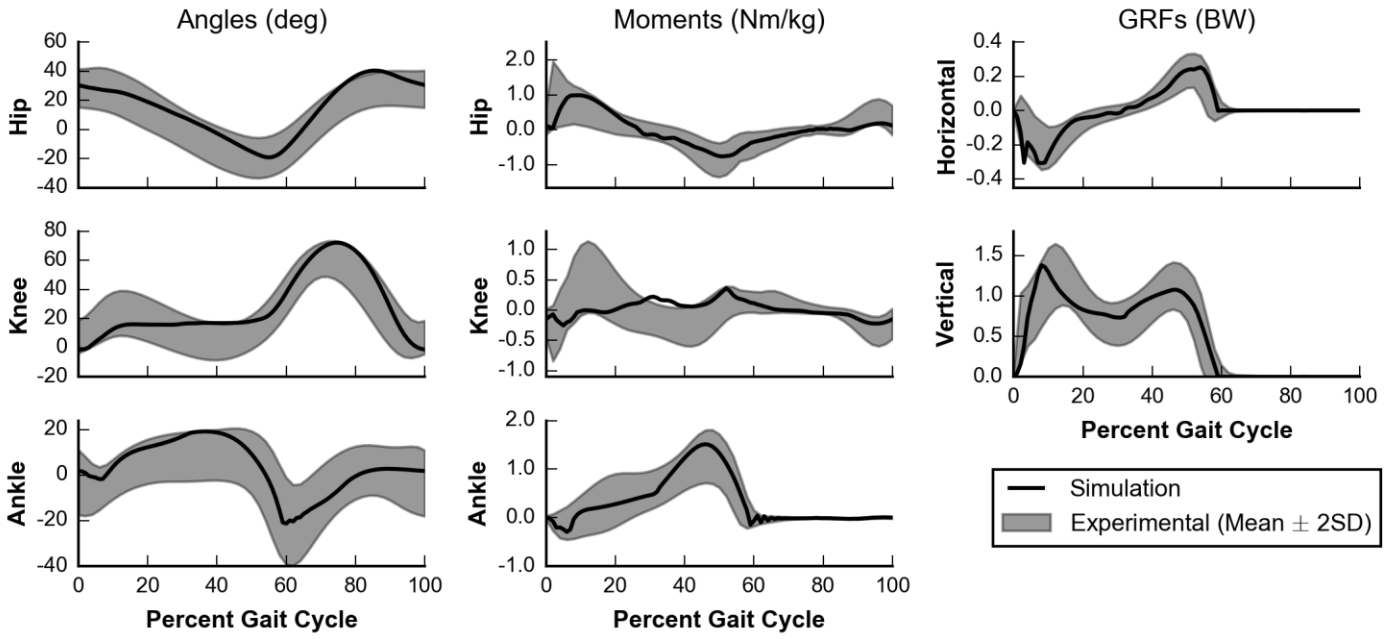

S1E Fig. Simulated walking at 1.46 m/s vs experimental data at 1.62 ± 0.18 m/s. To facilitate comparison, normalized speed from experimental data was converted using the model's leg length (0.856 m).

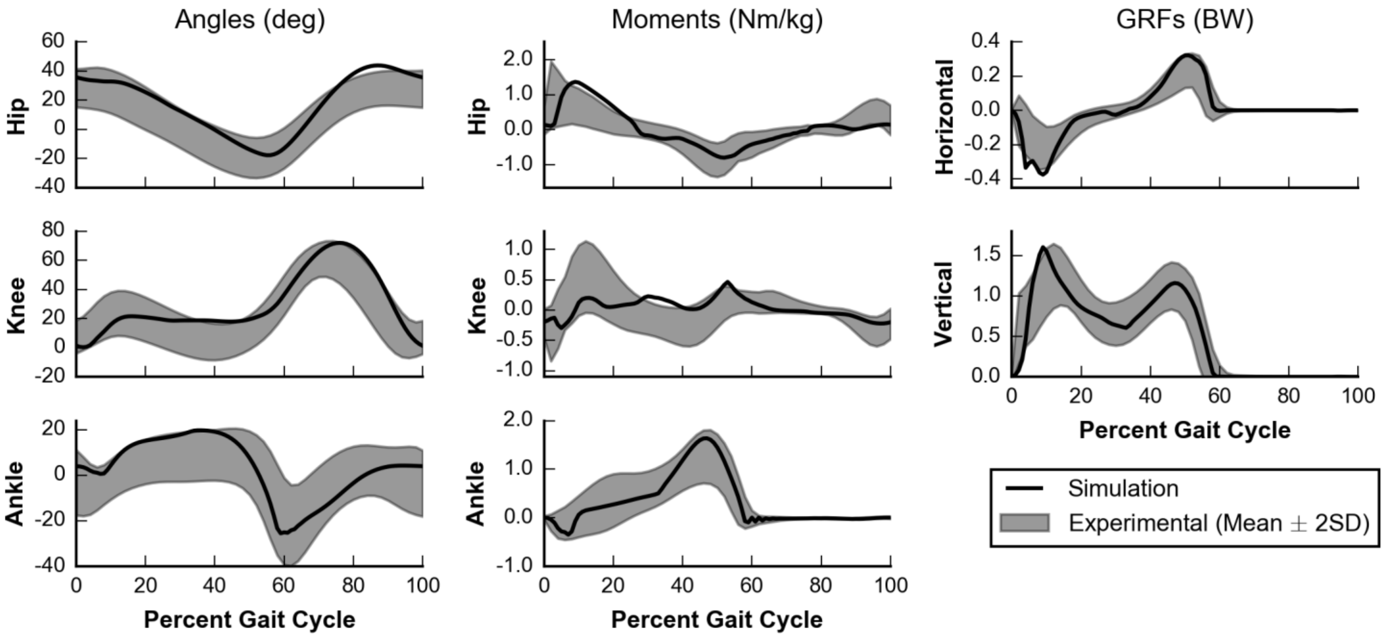

S1F Fig. Simulated walking at 1.71 m/s vs experimental data at 1.62 ± 0.18 m/s. To facilitate comparison, normalized speed from experimental data was converted using the model's leg length (0.856 m).

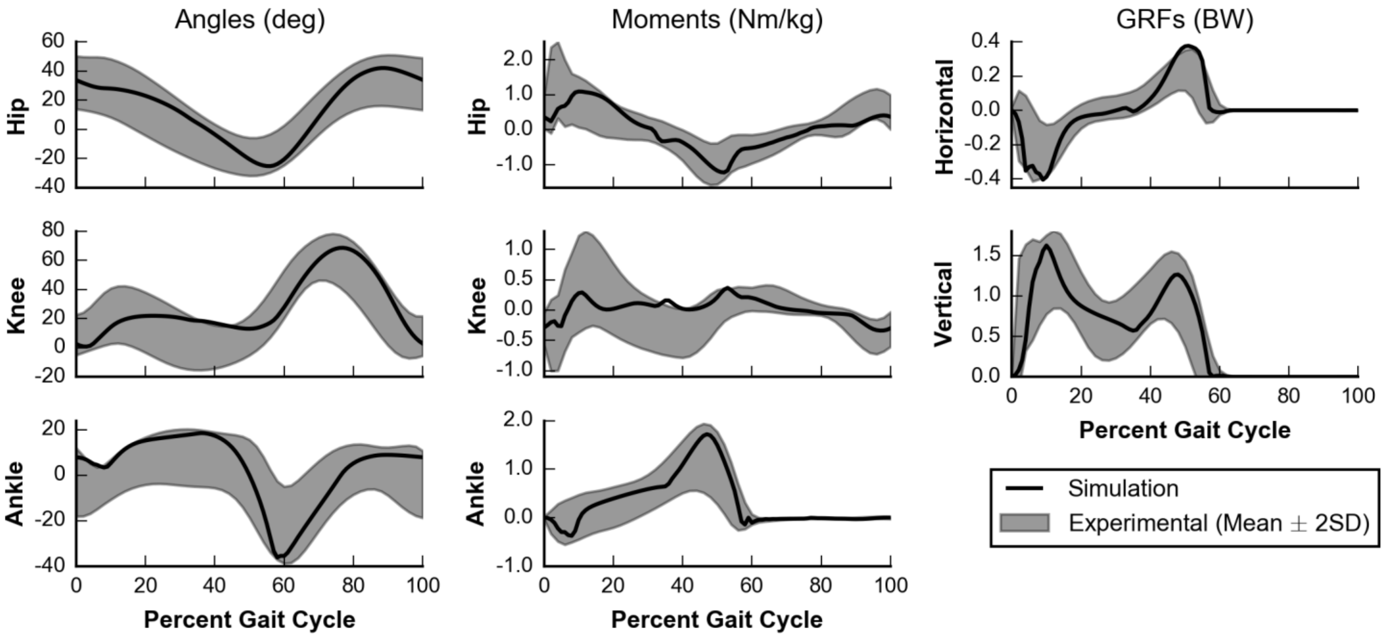

S1G Fig. Simulated walking at 1.96 m/s vs experimental data at 2.01 ± 0.27 m/s. To facilitate comparison, normalized speed from experimental data was converted using the model's leg length (0.856 m).
