# Supplementary material for: Predicting gait adaptations due to ankle plantarflexor muscle weakness and contracture using physics-based musculoskeletal simulations
Source: PLoS Comput Biol. 2019 Oct 7;15(10):e1006993. doi: 10.1371/journal.pcbi.1006993 (PMC6797212; doi:10.1371/journal.pcbi.1006993)
Supplement: S2 Fig — (PDF) [file pcbi.1006993.s002.pdf]

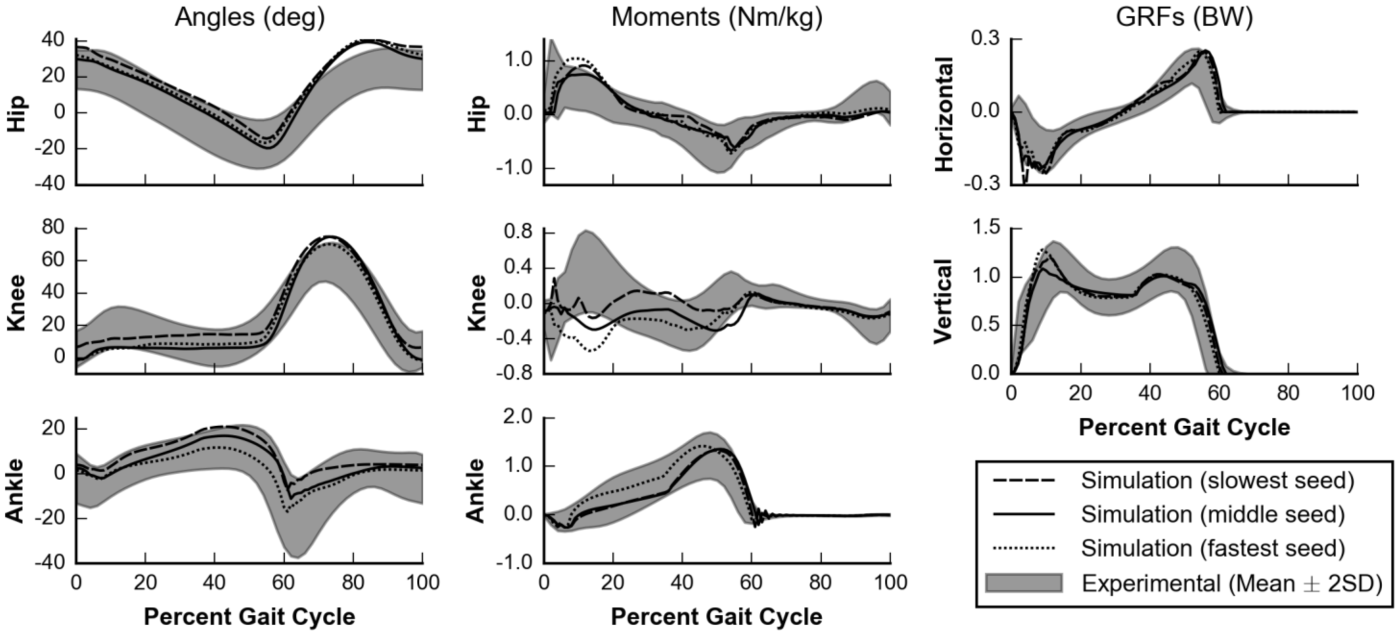

S2 Fig. Kinematic and kinetic trajectories for all three self-selected gait seeds (black lines) against experimental data (gray area).
