# Supplementary material for: Predicting gait adaptations due to ankle plantarflexor muscle weakness and contracture using physics-based musculoskeletal simulations
Source: PLoS Comput Biol. 2019 Oct 7;15(10):e1006993. doi: 10.1371/journal.pcbi.1006993 (PMC6797212; doi:10.1371/journal.pcbi.1006993)
Supplement: S4 Fig — (PDF) [file pcbi.1006993.s004.pdf]

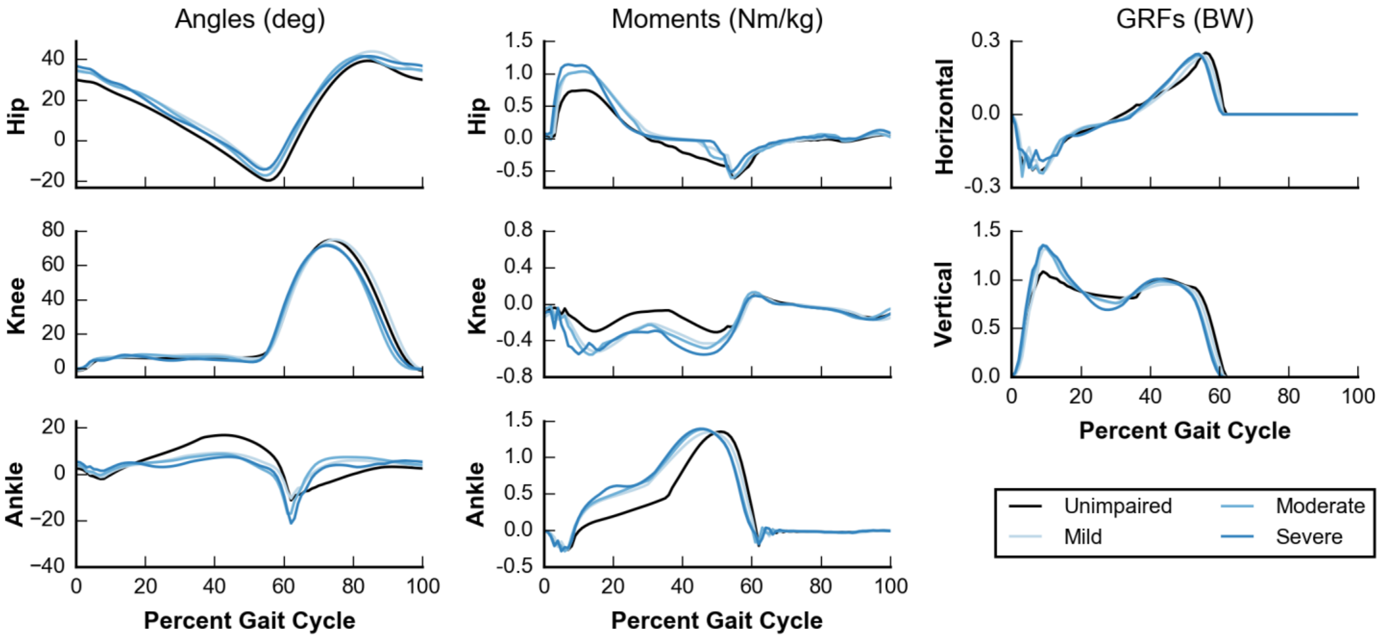

S4A Fig. Simulated walking with mild, moderate, and severe soleus (SOL) weakness.

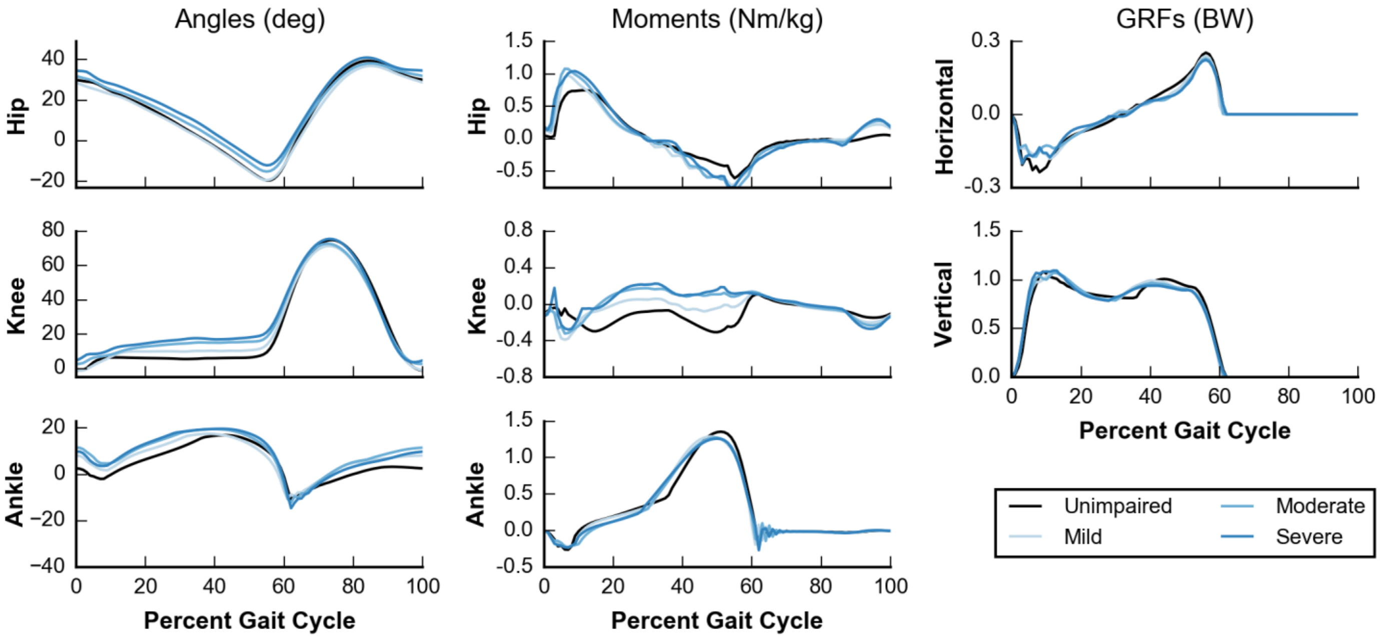

S4B Fig. Simulated walking with mild, moderate, and severe gastrocnemius (GAS) weakness.

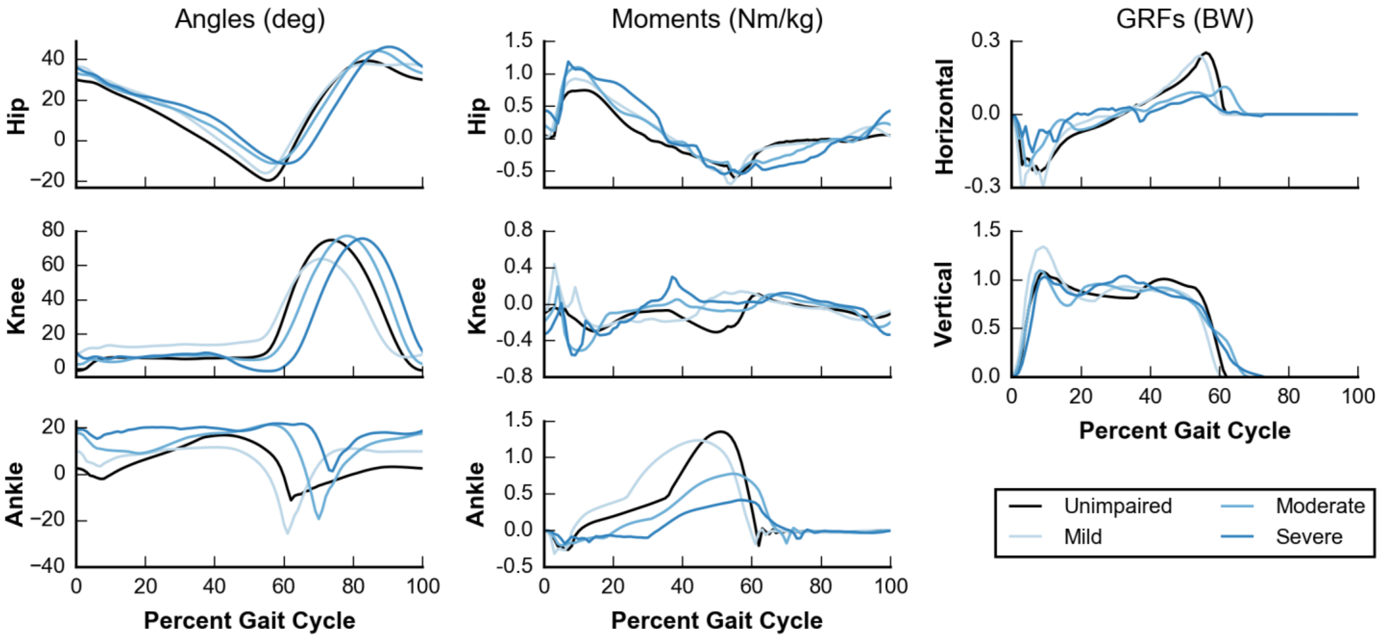

S4C Fig. Simulated walking with mild, moderate, and severe soleus and gastrocnemius (PF) weakness.

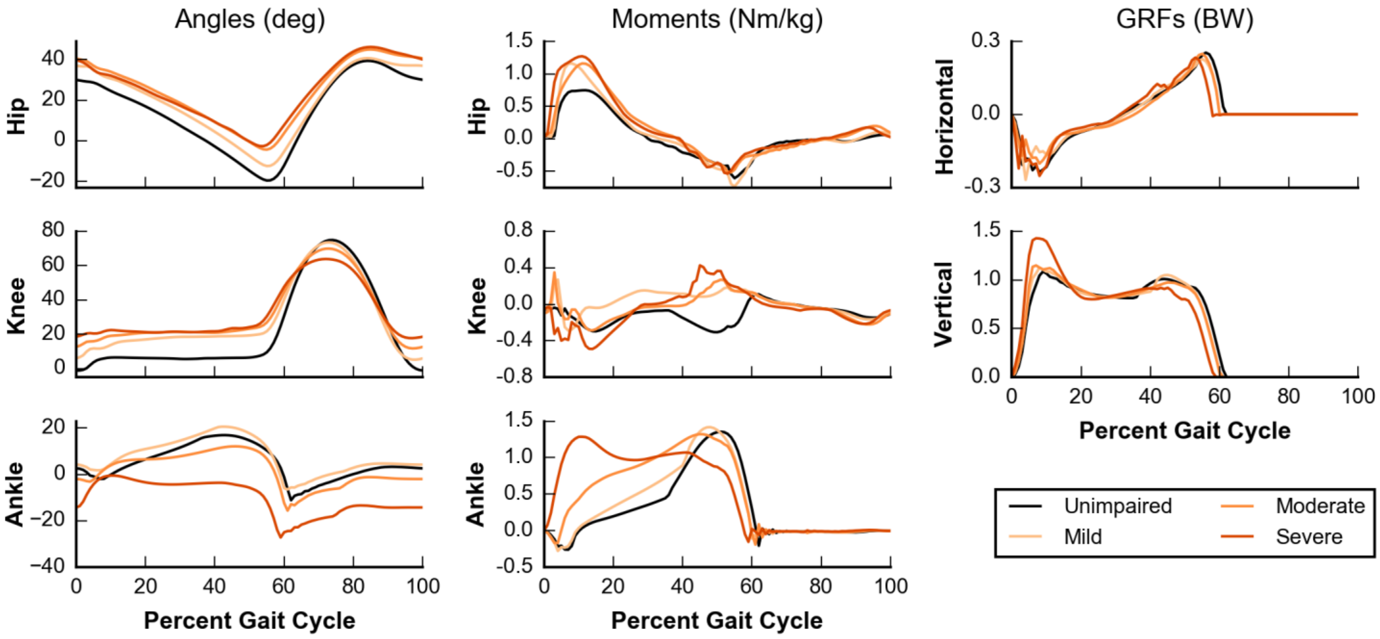

S4D Fig. Simulated walking with mild, moderate, and severe soleus (SOL) contracture.

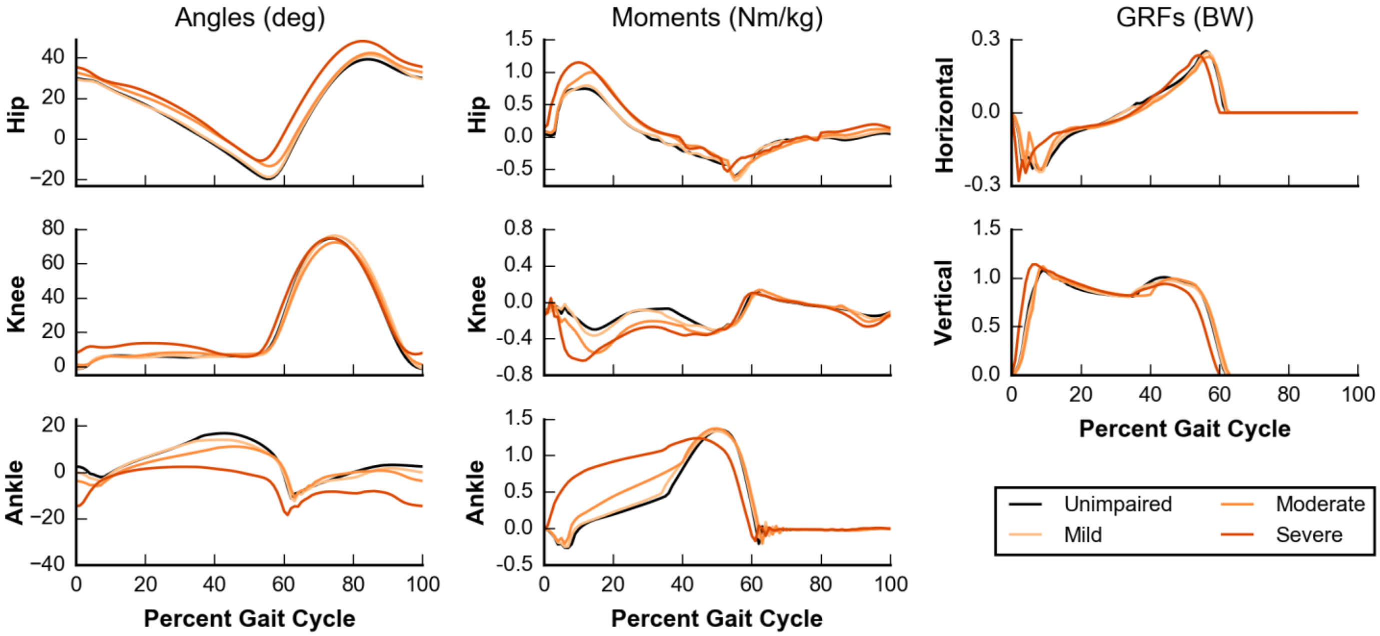

S4E Fig. Simulated walking with mild, moderate, and severe gastrocnemius (GAS) contracture.

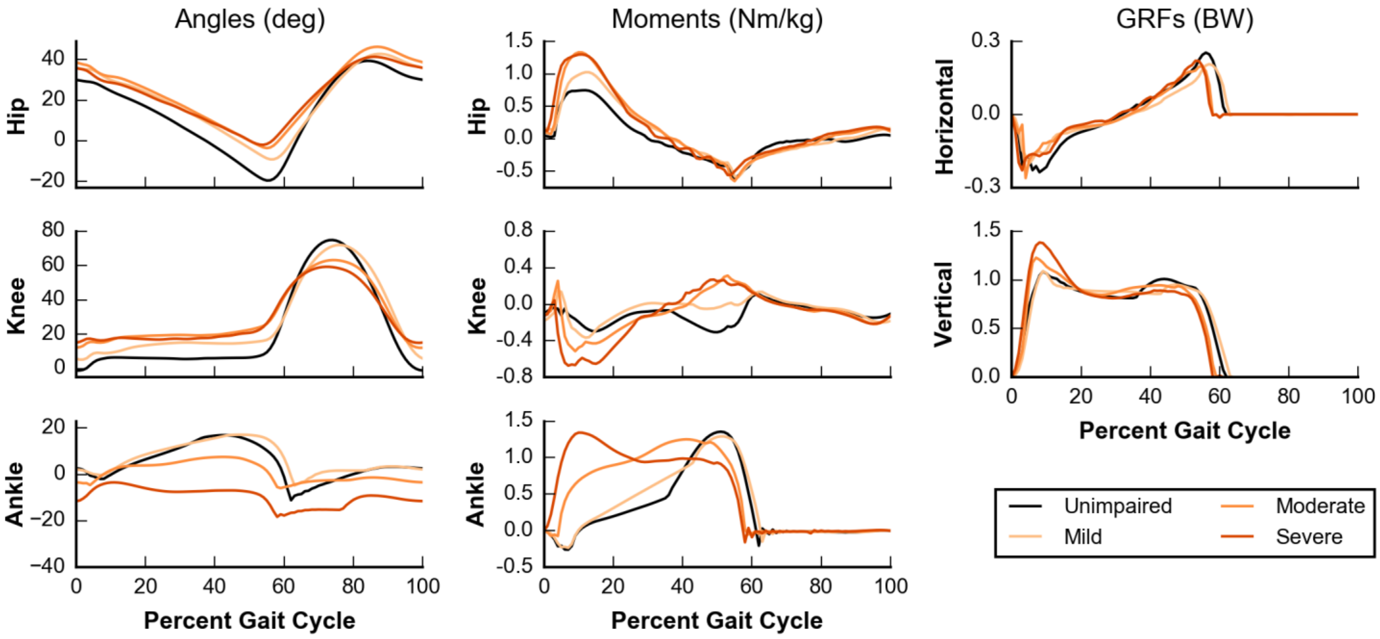

S4F Fig. Simulated walking with mild, moderate, and severe soleus and gastrocnemius (PF) contracture.
