# Supplementary material for: Predicting gait adaptations due to ankle plantarflexor muscle weakness and contracture using physics-based musculoskeletal simulations
Source: PLoS Comput Biol. 2019 Oct 7;15(10):e1006993. doi: 10.1371/journal.pcbi.1006993 (PMC6797212; doi:10.1371/journal.pcbi.1006993)
Supplement: S1 Table — (PDF) [file pcbi.1006993.s005.pdf]

S1 Table. Muscle parameters for the unimpaired musculoskeletal model.

Muscle name	Maximum isometric force (F_o^m)* [N]	Optimal fiber length (l_o^m) [m]	Muscle passive parameters (k^{PE}, ε_0^m)**	Maximum fiber contraction velocity [l_o^m/s]	Tendon slack length [m]	Tendon strain at F_o^m	Muscle path from *Delp et al. 1990* model
**ILPSO**	2697	0.117	(5, 0.6)	15	0.130	0.049	psoas
**GMAX**	3338	0.157	(5, 0.6)	15	0.048	0.049	gluteus maximus2
**RF**	2192	0.076	(9, 1.0)	15	0.346	0.049	rectus femoris
**HAMS**	4105	0.069	(5, 0.8)	15	0.349	0.049	semimembranosus
**VAS**	9594	0.099	(9, 1.0)	15	0.102	0.049	vastus intermedius
**BFSH**	557	0.11	(5, 0.6)	15	0.117	0.049	biceps femoris short head
**GAS**	4691	0.051	(5, 0.6)	15	0.384	0.1	medial gastrocnemius
**TA**	2117	0.068	(5, 0.6)	15	0.238	0.049	tibialis anterior
**SOL**	7925	0.044	(5, 0.6)	15	0.244	0.1	soleus

* Maximum isometric force is based on a specific tension of 60 N/cm^2.

** k^{PE} is the exponential shape factor for the passive force-length curve. ε_0^m is the passive muscle strain at F_o^m. The symbols match those from *Thelen 2003*.
