# Supplementary material for: Predicting gait adaptations due to ankle plantarflexor muscle weakness and contracture using physics-based musculoskeletal simulations
Source: PLoS Comput Biol. 2019 Oct 7;15(10):e1006993. doi: 10.1371/journal.pcbi.1006993 (PMC6797212; doi:10.1371/journal.pcbi.1006993)
Supplement: S2 Table — (PDF) [file pcbi.1006993.s006.pdf]

S2 Table. Initial Covariance Matrix Adaptation Evolutionary Strategy (CMA-ES) standard deviation for each free parameter.

Parameter	Parameter description*	Initial CMA standard deviation
K_C	constant excitation (Eq 1)	0.01
K_{L+}	length feedback gain (Eq 2)	0.1
l_o	length feedback offset (Eq 2)	0.05
K_{V+}	velocity feedback gain (Eq 3)	0.05
$K_{F\pm}$	force feedback gain (Eq 4)	0.1
K_p	pelvis tilt orientation feedback gain (Eq 5)	0.05
θ_o	pelvis tilt orientation offset (Eq 5)	0.01
K_v	pelvis tilt velocity feedback gain (Eq 5)	0.05
State transition: ES to MS	horizontal distance between ipsilateral foot and pelvis	0.01
State transition: PS to S	GRF on ipsilateral foot	0.01
State transition: S to LP	horizontal distance between ipsilateral foot and pelvis	0.01
State transition: LP to ES	GRF on ipsilateral foot	0.01
Initial positions	Initial pelvis tilt, hip, knee, and ankle angles	0.01
Initial velocities (except for pelvis horizontal velocity)	Initial pelvis tilt, hip, knee, and ankle angular velocities. Initial pelvis vertical translational velocity.	0.01
Initial pelvis horizontal velocity (targeted speed)**	Initial pelvis horizontal velocity during a simulation with a targeted speed.	0.01
Initial pelvis horizontal velocity (self-selected speed)**	Initial pelvis horizontal velocity during a self-selected speed simulation.	0.1

* Equation numbers in this column refer to those in the main text.

** Since these two affect the same parameter, only one of these is used depending on the type of simulation (i.e., targeted speed or self-selected speed).
