# Supplementary material for: Predicting gait adaptations due to ankle plantarflexor muscle weakness and contracture using physics-based musculoskeletal simulations
Source: PLoS Comput Biol. 2019 Oct 7;15(10):e1006993. doi: 10.1371/journal.pcbi.1006993 (PMC6797212; doi:10.1371/journal.pcbi.1006993)
Supplement: S3 Table — (PDF) [file pcbi.1006993.s007.pdf]

S3 Table. Muscle activity comparisons between simulations and *Perry and Burnfield, 2010* for Fig 7.

**Simulated muscle**	**Muscles from *Perry and Burnfield, 2010***
gluteus maximus (GMAX)	Gluteus Maximus, Upper<br>Gluteus Maximus, Upper
biarticular hamstrings (HAMS)	Biceps Femoris, Long Head<br>Semimembranosus<br>Semitendinosus*
iliopsoas (ILPSO)	Iliacus
rectus femoris (RF)	Rectus Femoris
vasti (VAS)	Vastus Intermedius<br>Vastus Lateralis<br>Vastus Medialis Longus<br>Vastus Medialis Oblique
biceps femoris short head (BFSH)	Biceps Femoris, Short Head*
gastrocnemius (GAS)	Gastrocnemius
soleus (SOL)	Soleus
tibialis anterior (TA)	Tibialis Anterior

* In *Perry and Burnfield, 2010*, "Semitendinosus" and "Biceps Femoris, Short Head" also have gray areas during late stance indicating that the activity was not seen in a majority of subjects. These are not included in Fig 7.
